# COVID-19-Related Psychosocial Care in General Hospitals: Results of an Online Survey of Psychosomatic, Psychiatric, and Psychological Consultation and Liaison Services in Germany, Austria, and Switzerland

**DOI:** 10.3389/fpsyt.2022.870984

**Published:** 2022-06-24

**Authors:** Rainer Schaefert, Barbara Stein, Gunther Meinlschmidt, Noa Roemmel, Christian G. Huber, Urs Hepp, Stéphane Saillant, Christian Fazekas, Frank Vitinius

**Affiliations:** ^1^Department of Psychosomatic Medicine, Faculty of Medicine, University Hospital Basel, University of Basel, Basel, Switzerland; ^2^Department of Psychosomatic Medicine and Psychotherapy, Paracelsus Medical Private University, Nuremberg General Hospital, Nuremberg, Germany; ^3^Division of Clinical Psychology and Epidemiology, Department of Psychology, University of Basel, Basel, Switzerland; ^4^Department of Clinical Psychology and Cognitive Behavioral Therapy, International Psychoanalytic University, Berlin, Germany; ^5^Department of Psychiatry (UPK), University of Basel, Basel, Switzerland; ^6^Integrated Psychiatric Services Winterthur – Zurcher Unterland, Winterthur, Switzerland; ^7^Department of General and Liaison Psychiatry, Neuchâtel Psychiatry Center (CNP), Neuchâtel, Switzerland; ^8^Department of Medical Psychology and Psychotherapy, Medical University of Graz, Graz, Austria; ^9^Department of Psychosomatics and Psychotherapy, Faculty of Medicine, University Hospital Cologne, University of Cologne, Cologne, Germany

**Keywords:** COVID-19, psychosocial care, general hospital, consultation and liaison service, psychosomatics, psychiatry, stress, staff support

## Abstract

**Background:**

The coronavirus disease 2019 (COVID-19) pandemic was accompanied by new challenges for psychosocial health care to enable the support of affected patients, their families, and staff in general hospitals. In this study, we aimed to describe the structures and procedures put in place by psychosomatic, psychiatric, and psychological consultation and liaison (CL) services in German, Austrian, and Swiss general hospitals, and to elucidate the emerging needs for cooperation, networking, and improvement.

**Methods:**

We conducted a cross-sectional online survey between December 2020 and May 2021, using a 25-item questionnaire derived from relevant literature, professional experience, and consultation with the participating professional societies. The survey was disseminated *via* national professional societies, relevant working and interest groups, and heads of the above-mentioned CL services.

**Results:**

We included responses from 98 CL services in the analyses, with a total response rate of 55% of surveyed hospital CL services; 52 responses originated from Germany, 20 from Austria, and 26 from Switzerland. A total of 77 (79%) of the 98 responding CL services reported that “COVID-19-related psychosocial care” (COVID-psyCare) was provided in their hospital. Among these, 47 CL services (61%) indicated that specific cooperation structures for COVID-psyCare had been established within the hospital. A total of 26 CL services (34%) reported providing specific COVID-psyCare for patients, 19 (25%) for relatives, and 46 (60%) for staff, with 61, 12, and 27% of time resources invested for these target groups, respectively. Regarding emerging needs, 37 (48%) CL services expressed wishes for mutual exchange and support regarding COVID-psyCare, and 39 (51%) suggested future changes or improvements that they considered essential.

**Conclusion:**

More than three-quarters of the participating CL services provided COVID-psyCare for patients, their relatives, or staff. The high prevalence of COVID-psyCare services targeting hospital staff emphasizes the liaison function of CL services and indicates the increased psychosocial strain on health care personnel during the COVID-19 pandemic. Future development of COVID-psyCare warrants intensified intra- and interinstitutional exchange and support.

**Trial Registration:**

ClinicalTrials.gov NCT04753242, version 11 February 2021.

## Introduction

The coronavirus disease 2019 (COVID-19) pandemic has been associated with significant psychosocial distress for patients and their relatives (1). Hospitalized COVID-19 patients experienced high rates of delirium, neuropsychiatric disorders, anxiety, depression, acute stress disorder (2) as well as posttraumatic stress disorder (PTSD) (3). Uninfected patients were also affected by the pandemic: Fear, isolation, and disengagement from care worsened premorbid mental disorders (2). Among relatives of patients hospitalized for COVID-19, 23% showed symptoms of psychological distress and 2% showed PTSD 30 days after hospital discharge (3). Prohibition of on-site visits was a special challenge, in particular, in end-of-life situations and case of death. Furthermore, caring for COVID-19 patients was related to the increased psychosocial burden of staff members in the hospital, including risks of becoming infected and of transmission of infection to family members and others, regular donning and doffing of personal protective equipment (PPE), shortage of PPE, exceptional workload, difficult treatment decisions, experience of unusually high numbers of patient deaths, rapidly changing information, reorganization factors, such as the deployment to new COVID-19 wards, and staff shortage (4). To summarize the burdens outlined for the different groups of affected persons, the COVID-19 pandemic is to be considered as a new form of trauma, and an urgent topic for psychosocial medicine (5, 6).

In Austria, Germany, and Switzerland, the damage caused by this first wave of the COVID-19 pandemic was managed relatively well, but the emergence of new and more virulent variant strains of the virus led to a devastating second wave of infections at the end of 2020 that resulted in far more hospitalizations and deaths in these countries, running into a subsequent third wave from March to May 2021 (7, 8). In affected regions worldwide, surveys indicated that 50% or more of physicians and nurses experienced clinically relevant levels of anxiety, depression, and acute stress disorder (2). A cross-sectional study about health care workers' (HCW) mental health during the first weeks of the COVID-19 pandemic in Switzerland reported that women (compared to men), nurses (compared to physicians), frontline staff (compared to non-front line workers), and HCWs exposed to COVID-19 patients (compared to non-exposed) reported more psychological symptoms than their peers (9). Comparably, a cross-sectional study in Germany found that the COVID-19 pandemic led to an increase in stress among HCWs (10). This situation raised new challenges for psychosomatic, psychiatric, and psychological consultation and liaison (CL) services in general hospitals worldwide in supporting COVID-19 patients, their relatives, and staff (2).

The definition of psychosomatic, psychiatric, and psychological CL services is structurally blurred and care structures vary between countries. However, these services typically deliver specialized mental health care for patients of general hospitals presenting with both physical and psychosocial health problems. They operate in somatic hospitals in a wide variety of medical settings, mainly on wards, but also in emergency units, and in outpatient clinics, including departments of internal medicine, geriatrics, oncology, surgery, and many more (11). In line with the bio-psycho-social model, they conduct a mix of consultation, liaison, specialized psychological interventions, training, and research. Usually, they have multidisciplinary staffing. Depending on local needs and circumstances, individual CL services vary widely (12); organizationally they are assigned to psychiatric, psychosomatic, or psychological departments. These services are vital in managing the interface between physical and mental health, and in training and supporting somatic hospital staff with regard to psychosocial issues (13). In contrast to many other countries, in Germany, Psychosomatic Medicine is not a synonym for consultation-liaison psychiatry but represents a comprehensive field as well as a specialized medical discipline (14). Hence, Psychosomatic Medicine in Germany has a larger institutional basis than in many other countries. As a core task, in Germany, Departments of Psychosomatic Medicine at somatic hospitals provide a psychosomatic CL service for the entire hospital, usually in addition to the psychiatric CL service.

During the first wave of the COVID-19 pandemic, the speakers of the working group on consultation and liaison psychosomatics of the German College of Psychosomatic Medicine (DKPM) and the German Society for Psychosomatic Medicine and Medical Psychotherapy (DGPM) entered into an online exchange in order to support each other and to discuss following questions: How do the different CL services deal with the COVID-19 situation at the different hospitals and what can be learned from another? This exchange led to an online survey of psychosomatic, psychiatric, and psychological CL services in somatic hospitals in Germany, Austria, and Switzerland during the second and third waves of the COVID-19 pandemic. The goal was to get to know the situation across CL services and to allow profiting from reported experiences. Our project followed a call for action for mental health research efforts for the COVID-19 pandemic published in Lancet Psychiatry in April 2020 (15). It is in line with the recommendations of the Report of the Academy of Consultation-Liaison Psychiatry Task Force on Lessons Learned From the COVID-19 Pandemic, published in July 2021 (2). CL services were challenged to adapt during the pandemic, possibly bringing permanent changes to our profession (16). The overarching objective of our study was to summarize the efforts made in “COVID-19-related psychosocial care” (COVID-psyCare) in general hospitals and to build upon the experience gained so far, to optimize response to the current pandemic and future pandemics. In this context, the aims of this study were:

(1) to describe the COVID-psyCare structures put in place by psychosomatic, psychiatric, and psychological CL services,
(2) to review specific services aimed at patients, relatives, and staff, and
(3) to elucidate emerging needs for cooperation, networking, and improvements.

## Materials and Methods

### Study Design and Ethical Approval

This health services research project was carried out as an observational study in the form of a cross-sectional online survey in Germany, Austria, and Switzerland. The study was led by the spokespersons of the working group consultation and liaison psychosomatics of DKPM and DGPM. The survey was performed and sent out together with the respective national societies from Germany (DKPM and DGPM/Chief Physician Conference of Psychosomatic-Psychotherapeutic Hospitals and Departments, CPKA), Austria (Austrian Society for Psychosomatics and Psychotherapeutic Medicine, ÖGPPM), and Switzerland (Swiss Academy of Psychosomatic and Psychosocial Medicine, SAPPM/ Association of Psychosomatic Chief Physicians, and Swiss Society of Consultation-Liaison Psychiatry and Psychosomatics, SSCLPP). We formed a steering group with representatives from Germany (BS, FV), Austria (CF), and Switzerland (RS, CH). We obtained written statements, declarations, or votes from the responsible ethic committees in Cologne (Ethics Committee of the Medical Faculty of the University of Cologne, 20-1416_1), Graz (Ethics Committee of the Medical University of Graz; 33-120ex 20/21), and Basel (Ethics Committee of Northwest and Central Switzerland, EKNZ, Req-2020-00861). Participation in the survey was voluntary. We obtained written informed consent from each participant before responding to the survey. Participants could cancel the survey at any time and without giving reasons. The study was registered on ClinicalTrials.gov (NCT04753242, version 11 February 2021).

### Setting and Participants

This study was an online survey aiming at psychosomatic, psychiatric, and psychological CL services at general hospitals in Germany, Austria, and Switzerland. We reached out to the representatives of the services *via* the respective national professional societies and relevant working and interest groups (see above).

The number of psychosomatic, psychiatric, and psychological CL services is not known in most countries worldwide; the same is true for Germany, Austria, and Switzerland. Nevertheless, together with the participating professional societies, we tried to get as good an estimate as possible of the number of services to which the survey was sent out. For Germany, the number of CL services in general hospitals with a Psychosomatic Department was estimated at 74 according to the Directory of German Hospitals, the number of CL services in Austria was estimated at 55, and in Switzerland, there was an estimated number of 50 psychosomatic, psychiatric, and psychological CL services. We aimed to contact the heads of these services *via* email to ask for their participation in the survey. Emails were sent out *via* email distribution lists of the national societies, complemented by individual email contacts. One to two reminder emails were sent *via* these lists. We asked the CL services to assign one representative to reply to the survey. The online survey was open from December 2020 to May 2021.

**Figure 1:**
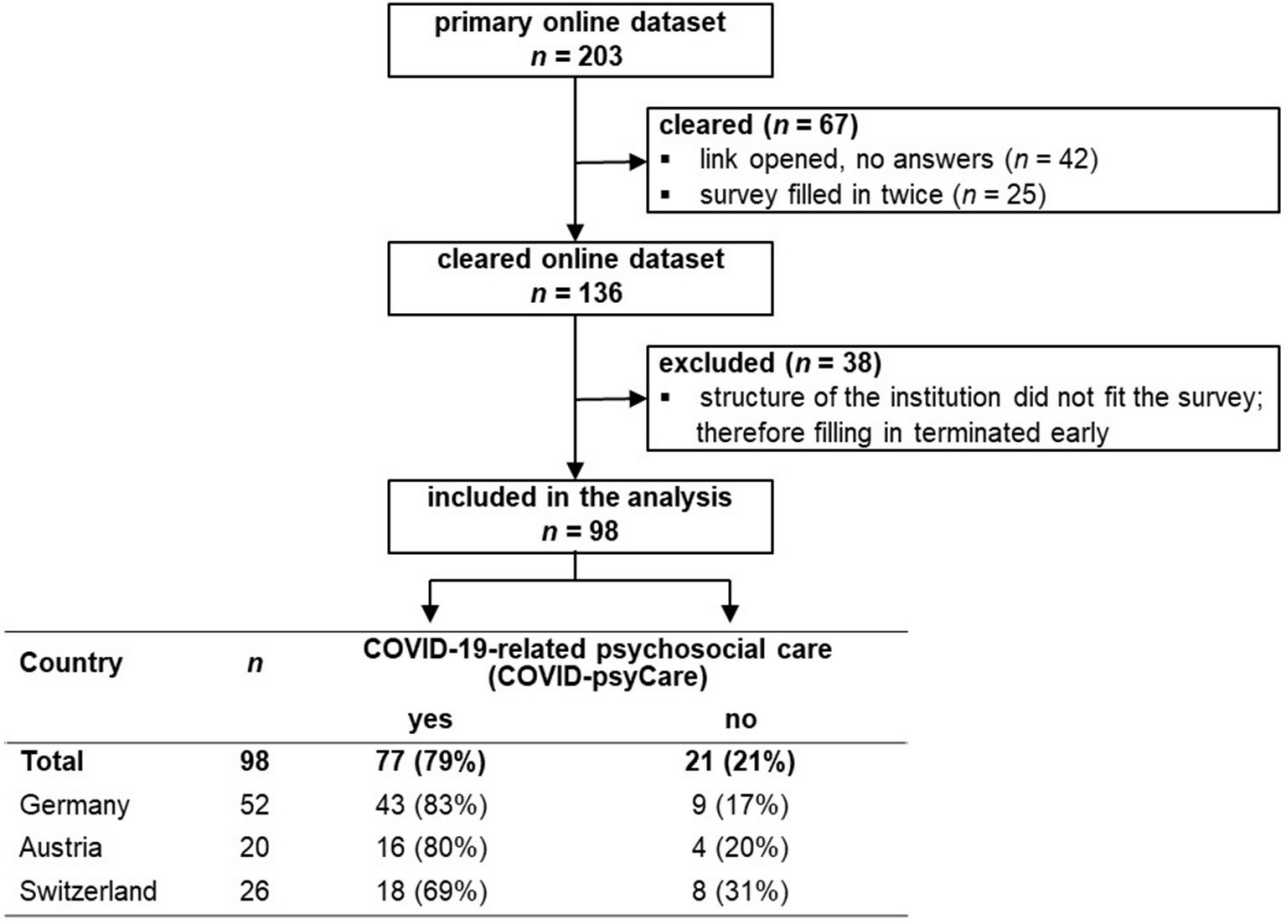
Study flow chart.

As shown in Figure 1, the dataset was cleared from records with the description “link opened, no answers” (*n* = 42), if the link was only opened, but no information was entered into the survey. In case the questionnaire was filled in twice by the same CL service, the most complete record was taken (*n* = 25). Furthermore, records were excluded from the analyses if the structure of the institution did not fit the survey, and therefore filling in usually was terminated during the characterization of the service in the course of the first six questions (*n* = 38).

### Study Questionnaire and Outcome Measures

We used a self-developed questionnaire based on relevant literature (9, 15), expert experience, and consultation with participating professional societies. The survey contained 25 questions on structural and process variables regarding somatic and psychosocial care structures, services, and procedures that psychosocial CL services have established for patients, their relatives, and staff in general hospitals in the context of the COVID-19 pandemic, as well as needs and requests for the future:

(1) Characterization of participating hospitals as well as somatic and psychosocial care structures:
  ○ Characteristics of the hospital: type of hospital, number of beds of the hospital.
  ○ Characteristics of somatic care: Involvement in the somatic care of COVID-19 patients and the extent the hospitals were maximally occupied regarding the somatic care of COVID-19 patients, both measured with a 6-point Likert scale from 0 = “not at all” to 5 = “very strongly”; structures developed for the somatic care of COVID-19 patients: Wards and units for the treatment of COVID-19 patients as well as special structures for somatic care related to COVID-19 established in the hospitals.
  ○ Characteristics of psychosocial care: Psychosocial services available in the hospital; professional perspective this survey was answered from; COVID-psyCare established in the hospital; partners involved in cooperation structures for COVID-psyCare; established structures for COVID-psyCare; psychosocial representative in the COVID-19 task force; maximum availability of psychosocial care for COVID-19 patients in terms of time.
(2) Implementation and use of specific services or procedures of COVID-psyCare for the three target groups patients, their relatives, and hospital staff: Evaluation of the extent to which these services and procedures have proven successful on a 6-point Likert scale from 0 = “not at all” to 5 = “very strongly”; ways of communication with the different target groups about COVID-psyCare services.
(3) Maximum COVID-19 related burden of the psychosocial teams measured on a 10-point Likert scale from 1 = “not stressed at all” to 10 = “extremely stressed”; needs and requests for the future: Specification of required exchange/support as well as of changes/improvements that are considered essential for the future concerning psychosocial care services in ones hospital in the COVID-19 context.

The representatives of the steering group for Germany, Austria, and Switzerland reviewed the questionnaire and ensured that all specific national aspects were covered. For Switzerland, the study questionnaire was translated by a professional company from German into French and into Italian and proofread by CL employees proficient in French and Italian, respectively. Participants were free to choose the language version to reply to. We provide an English version and the original German version of the questionnaire as Supplementary Material to this article.

### Data Management

All collected data were pseudonymized before processing. Data collection was carried out with the online survey tool Questback EFS Fall 2019/license model “Unipark” of Questback GmbH *via* the University of Basel. Questback stores the data collected *via* the tool in the server park in Frankfurt/Main. This is reliably protected from external access. The BSI-certified data center is subject to high data protection and security requirements according to ISO 27001 based on “IT-Grundschutz.” Subsequently, we stored, processed, and analyzed the data at the University Hospital Basel. Further processing of the anonymized data and interpretation of the results were carried out in cooperation with the German, Austrian, and Swiss members of the above-mentioned steering group.

### Statistical Methods

All analyses were conducted using the statistical software package IBM SPSS Statistics Version 25. Missing data were not imputed. Results were analyzed using descriptive statistics. Numbers and percentages were used to present the data. Here, the prevalence was presented for categorical variables, means, and standard deviations for continuous variables.

## Results

### Study Response

We provide the study flow chart in Figure 1. Altogether, we had an initial set of *n* = 203 responses from Germany, Austria, and Switzerland. A total of 67 responses had to be cleared: *n* = 42 were records with only missings, indicating that the link was only opened, but none of the questions of the survey was answered; *n* = 25 were filled in twice by the same CL service; in this case, the most complete record was taken. Furthermore, *n* = 38 records had to be excluded from the analyses because the structure of the institution did not fit the survey, and therefore filling in usually was terminated during the characterization of the service in the course of the first six questions. This led to a final dataset of *n* = 98 responses that could be included in the analyses.

### Characterization of Participating Hospitals and CL Services

Table 1 shows the baseline description of the *n* = 98 CL services included in the analyses. Thus, data were available from 55% of CL services based on an estimated denominator of 179 CL services in total as described above. A total of 52 responses originated from Germany (data available from 70% of the CL services), 20 from Austria (data available from 36% of the CL services), and 26 from Switzerland (data available from 52% of the CL services). We provide information on further characteristics of these services, including the type of hospital, psychosocial services available in the hospital, and the professional perspective this survey was answered from in Table 1. About psychosocial services available in the hospital, there were 19 entries of “other services” available in addition to the classic ones (e.g., Social service, Psycho-oncology, Pastoral care, Child and adolescent psychiatry, and Psychosomatics). About the professional perspective this survey was answered from, there were 10 entries of “other perspectives” (Psycho-oncology, Pain therapy, Psychotherapy, Internal medicine, Geriatrics, Gynecology) in addition to the classic ones. Typically, CL services were staffed multidisciplinary on average consisting of 1.4 full-time equivalents (FTE) physicians, 1.7 FTE psychologists, 0.2 FTE social workers, 0.2 FTE nursing personnel, and 0.1 FTE other positions (see Table 1).

**Table 1:** Sample descriptive.

	**Total**	**Germany**	**Austria**	**Switzerland**
	**(*n* = 98)**	**(*n* = 52)**	**(*n* = 20)**	**(*n* = 26)**
**Estimated overall number of respective CL services**	179	74*	55	50
**Data available from CL services**	55%	70%	36%	52%
**Type of hospital;** ***n*** **(%)**				
University hospital	29 (30%)	18 (35%)	7 (35%)	4 (15%)
General hospital	44 (45%)	28 (54%)	10 (50%)	6 (23%)
Specialized hospital	15 (15%)	5 (10%)	1 (5%)	9 (35%)
Other type of hospital	10 (10%)	1 (2%)	2 (10%)	7 (27%)
**Number of beds of the hospitals**				
Number of beds of the hospital; mean (SD; 95% CI)	671 (632; 545–798)	863 (656; 681–1,046)	750 (645; 448–1,052)	227 (262; 120–333)
0–299 beds; *n* (%)	34 (35%)	7 (14%)	7 (35%)	20 (77%)
300–599 beds; *n* (%)	24 (25%)	17 (33%)	4 (20%)	3 (12%)
> =600 beds; *n* (%)	40 (41%)	28 (54%)	9 (45%)	3 (12%)
**Psychosocial services available in the hospital (multiple answers possible);** ***n*** **(%)**				
Psychosomatic CL service	68 (69%)	48 (92%)	6 (30%)	14 (54%)
Psychiatric CL service	68 (69%)	38 (73%)	15 (75%)	15 (58%)
Psychological CL service	40 (41%)	16 (31%)	16 (80%)	8 (31%)
Other psychosocial services	19 (19%)	9 (17%)	3 (15%)	7 (27%)
**Professional perspective this survey was answered from (multiple answers possible);** ***n*** **(%)**				
Psychosomatic Medicine	71 (72%)	45 (87%)	10 (50%)	16 (62%)
Psychiatry	25 (26%)	7 (13%)	7 (35%)	11 (42%)
Psychological service/Psychological Department	11 (11%)	1 (2%)	8 (40%)	2 (8%)
Medical psychology	6 (6%)	2 (4%)	4 (20%)	0
Child & adolescent psychiatry & psychosomatics	1 (1%)	0	1 (5%)	0
Other	10 (10%)	3 (6%)	4 (20%)	3 (12%)
**Full-time equivalents in consultation-liaison services; mean (SD; min-max)**				
Physician positions	1.4 (1.4; 0–5.8)	1.3 (1.3; 0–5.0)	1.5 (1.6; 0–5.8)	1.5 (1.5; 0–4.0)
Psychologist positions	1.7 (2.3; 0–8.85)	1.5 (2.2; 0–8.9)	1.3 (1.9; 0–6.0)	2.9 (3.0; 0–8.0)
Social worker positions	0.2 (0.6; 0–4.0)	0.2 (0.6; 0–4.0)	0.3 (0.5; 0–1.6)	0.2 (0.4; 0–1.0)
Nursing positions	0.2 (0.5; 0–2.5)	0.9 (0.4; 0–2.0)	0.4 (0.7; 0–2.5)	0.2 (0.5; 0–2.0)
Other positions	0.1 (0.4; 0–2.0)	0	0.3 (0.6; 0–2.0)	0.1 (0.4; 0–1.0)

^*^CL services in general hospitals with a Psychosomatic Department according to the Directory of German Hospitals.

### Somatic Care of COVID-19 Patients: Involvement and Structures

**Figure 2:**
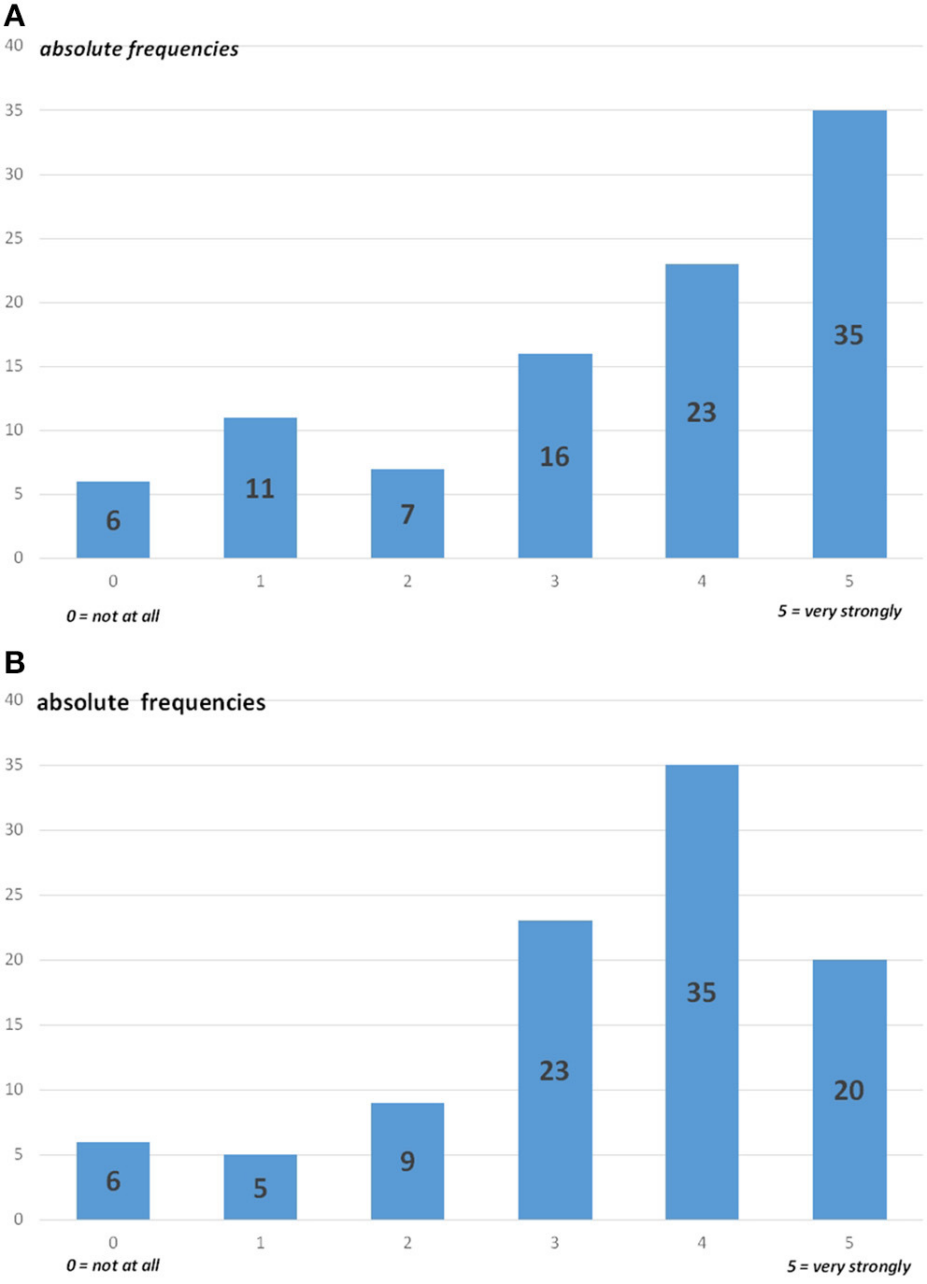
Involvement in the somatic care of COVID-19 patients. **(A)** Maximum level of involvement of the hospitals (*n* = 98) in the somatic care of COVID-19 patients since the beginning of the pandemic. **(B)** Extent the hospitals (*n* = 98) were maximally occupied regarding the somatic care of COVID-19 patients.

This paragraph shows the somatic care of COVID-19 patients established by the 98 hospitals with participating CL services in this survey. Figure 2 depicts the maximum level of involvement of the hospital in the somatic care of COVID-19 patients since the beginning of the pandemic (Figure 2A) and the extent to which hospitals were maximally occupied regarding the somatic care of COVID-19 patients (Figure 2B).

**Figure 3:**
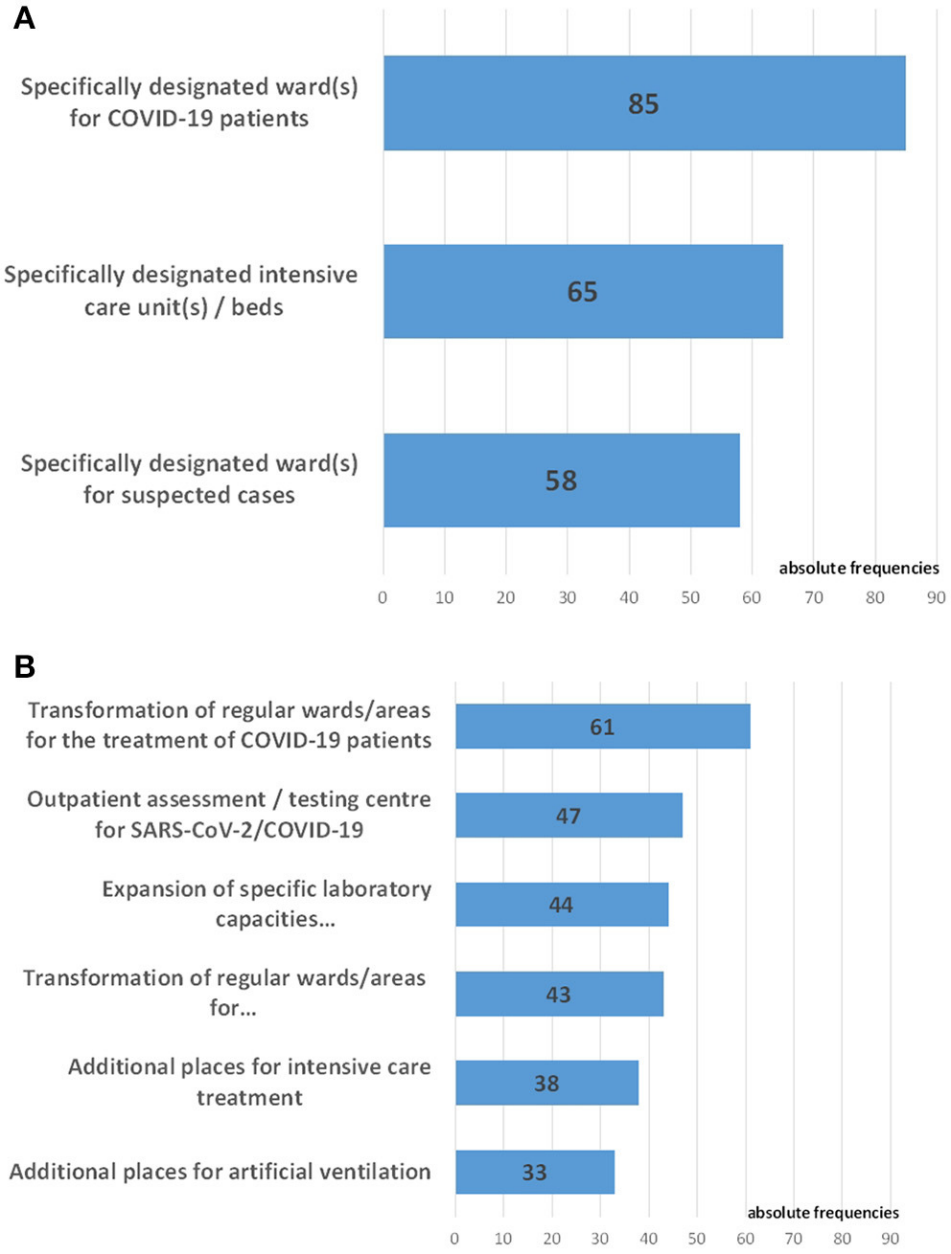
Structures developed for the somatic care of COVID-19 patients. **(A)** Hospital wards and units for the treatment of COVID-19 patients (*n* = 98). **(B)** Special structures for somatic care related to COVID-19 established in the hospitals (*n* = 98).

Figure 3 shows the hospital wards and units where COVID-19 patients were treated as inpatients (Figure 3A) as well as newly established special hospital structures for somatic care related to COVID-19 (Figure 3B).

### COVID-psyCare in Somatic Hospitals: Establishment and Structures

A total of 77 of the 98 CL services (79%) reported that in their hospital psychosocial care was provided in connection with COVID-19, whereas 21 CL services (21%) provided no COVID-psyCare. The following information refers to those 77 CL services (43 from Germany, 16 from Austria, 18 from Switzerland) that offered COVID-psyCare.

**Figure 4:**
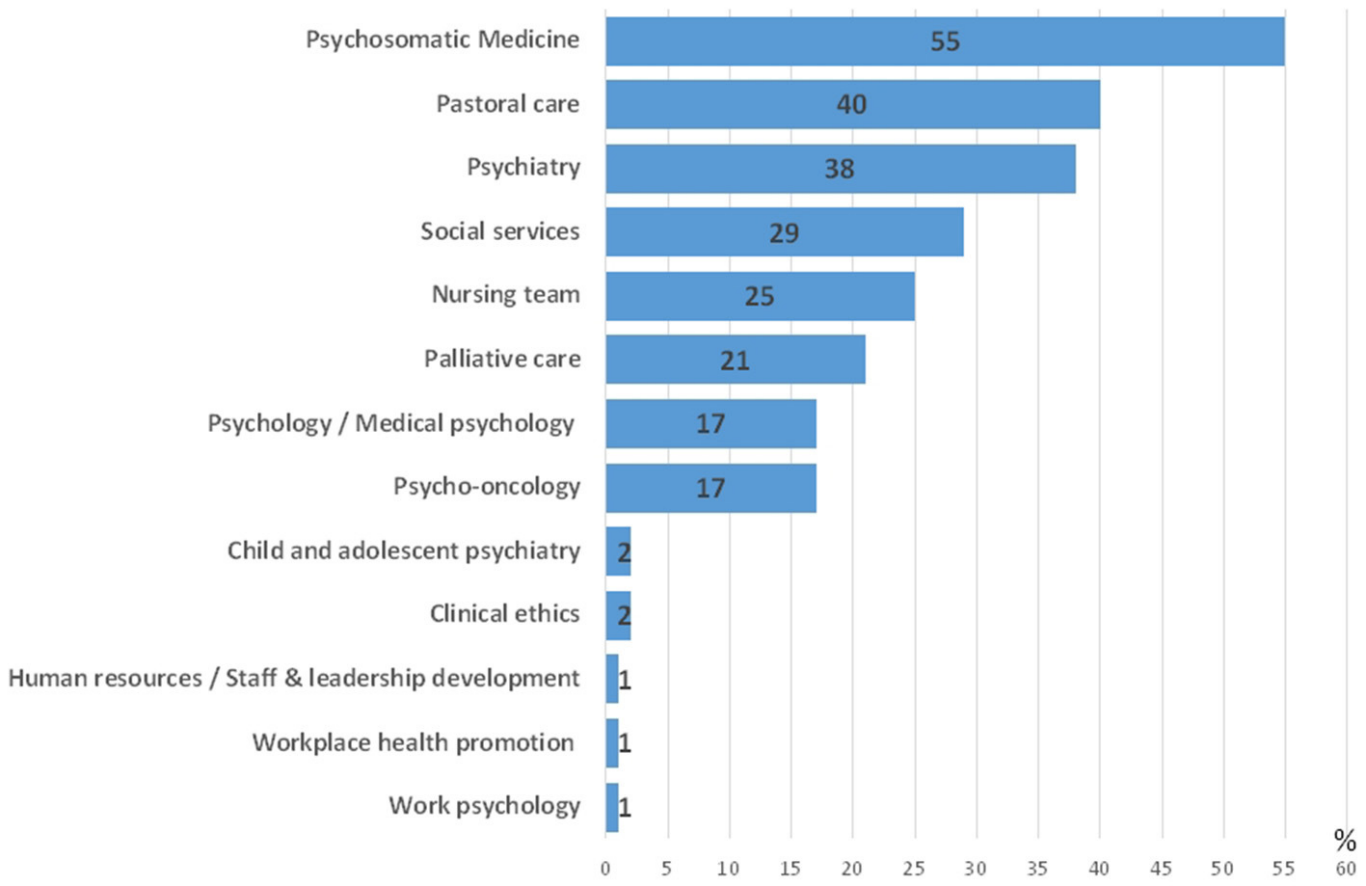
Partners involved in cooperation structures for COVID-psyCare (relative frequencies, *n* = 77 CL services with COVID-psyCare, multiple answers possible).

Among these 77 CL services, 47 (61%) answered that additional cooperation structures had been established within the hospital for psychosocial support in the context of the COVID-19 pandemic, 25 (33%) CL services reported no such additional structures, while 5 (6%) CL services did not answer this question. The partners involved in these cooperation structures are depicted in Figure 4.

Regarding the established structures (multiple answers possible), 44 (57%) of the 77 CL services providing COVID-psyCare stated that existing care structures were refined, 26 (34%) had instituted new care structures, and 17 (22%) reported that their care structure had remained unchanged. Some CL services also commented on which structures for COVID-psyCare had been developed. These were special structures to support staff: regular team meetings in the COVID intensive care unit (ICU), and as needed, also on other wards as well as telephone hotlines for employees. During home-office due to lockdown, treatment was also conducted *via* telephone. Temporarily overlapping structures were created, which then dissolved again.

Among the 77 CL services providing COVID-psyCare, 32 (42%) reported that in the COVID-19 task force of the hospital a specific representative had been appointed for psychosocial issues, 38 (49%) denied, seven (9%) did not answer that question. Thirteen (17%) CL services reported that a responsible person from the psychosocial departments regularly participated in the task force, 16 (21%) stated that this person selectively participated in the task force on demand. Three (4%) CL services reported the delegation of a responsible person outside the psychosocial departments with regular participation, 2 (3%) with selective participation in the task force meetings. One comment on this question stated that contact was made on demand by the COVID task force.

**Figure 5:**
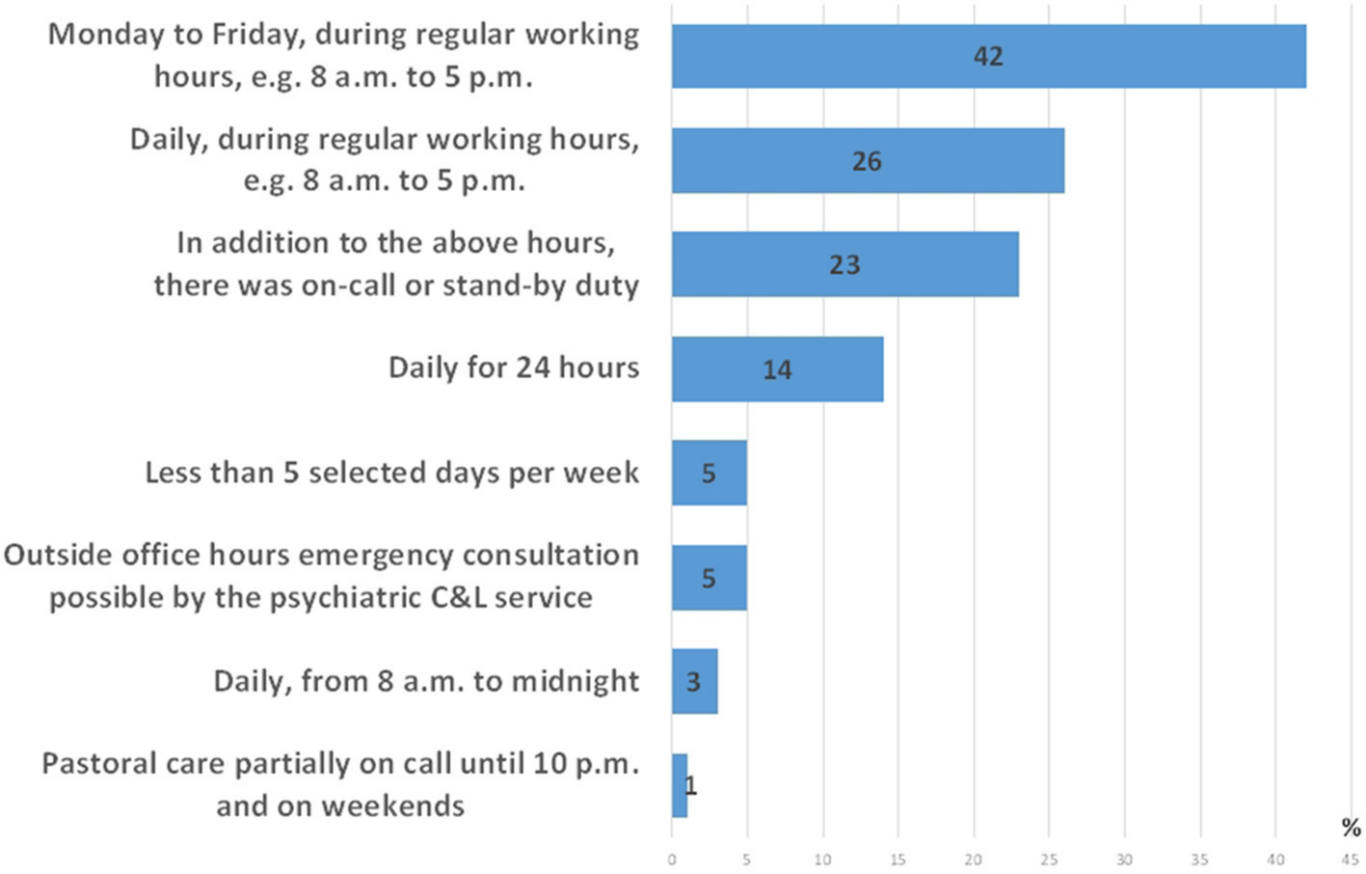
Maximum availability of psychosocial care for COVID-19 patients in terms of time (multiple answers possible).

The maximum availability of psychosocial care for COVID-19 patients in terms of time (multiple answers possible) is depicted in Figure 5.

### Specific Services or Procedures of COVID-psyCare

Table 2 shows the specific services or procedures of COVID-psyCare for the three target groups. Of the 77 CL services that additionally provided COVID-psyCare, 26 (34%) reported the provision of interventions for COVID-19 patients, 19 (25%) reported services for relatives of COVID-19 patients, and 46 (60%) reported additional COVID-19-related services for staff. Regarding the percentage of time utilization of the CL mental health team by COVID-psyCare, 61% of the time was spent on patient care, 12% on relatives, and 27% on staff. The specific services or procedures for the three target groups are depicted in Table 2.

**Table 2:** Specific services or procedures of COVID-psyCare offered by the CL services providing COVID-psyCare (*n* = 77).

**Specific services or procedures …**	**Number of** **CL services** (n^*^ = 77)	**Percentage of time utilization mean** **(min - max; SD)**
**… for patients**	26 (34%)	61% (0 - 100; 27)
**… for relatives**	19 (25%)	27% (0 - 70; 13)
**…. for staff**	46 (60%)	12% (0 - 100; 25)
**Specific services or procedures …**	***n*** *	**Extent to which these procedures/offers have proven successful** **(0 = not at all to** **5 = very strongly)** **mean** **(min–max; SD)**
**… for patients**		
COVID-19 telephone hotline	16	3.00 (0–5; 1.75)
Consultation staff specifically for COVID-19 referrals	16	3.69 (2–5; 1.01)
Specific protocols/SOPs for common questions	12	3.75 (2–5; 0.87)
Psychosocial COVID-19 Care Team	11	3.18 (0–5; 1.72)
Liaison staff on COVID wards	11	4.00 (1–5; 1.34)
Aftercare services for patients with post-COVID syndrome	9	3.00 (1–5; 1.12)
COVID-19 outpatient clinic	6	1.17 (0–5; 2.04)
Others	4	3.75 (1–5; 1.89)
**… for relatives**		
COVID-19 telephone hotline also for relatives	13	3.08 (2–5; 1.67)
Specific counseling for relatives	13	3.69 (2–5; 1.82)
Specific protocols/SOPs for supporting relatives	7	3.71 (2–5; 1.11)
Others	3	3.33 (1–5; 2.08)
**… for staff**		
Telephone hotline for staff	32	2.47 (0–5; 1.50)
Case discussions on patient-related stressful situations	28	3.64 (0–5; 1.42)
Team supervision/facilitated group exchange on how the corona situation is experienced as staff and in the team	25	3.67 (1–5; 1.20)
Workshops to strengthen the resilience of staff (e.g., self-care/resource activation)	12	2.33 (0–5; 1.61)
Creating relaxation opportunities for teams under high stress levels	9	2.67 (0–5; 1.58)
Targeted work with team leaders/supervisors on helpful support measures for staff/teams	8	3.38 (1–5; 1,60)
Training in dealing with psychosocial stress of patients and relatives (recognition, communication, management)	8	2.75 (0–5; 1.98)
Others	6	3.00 (1–5; 1.27)

*Absolute frequencies*.

Ways of communication with the different target groups regarding the COVID-psyCare services are depicted in Table 3.

**Table 3:** Ways of communication with the different target groups about COVID-psyCare services (absolute frequencies).

	**Patients**	**Relatives**	**Staff**
**In person**	31	27	30
**Word-of-mouth recommendation**	15	14	28
**Internet**	11	13	39
**Information** ***via*** **senior executives**	–	–	29
**Flyer**	9	4	15
**Notice board**	3	0	13
***Via*** **the nursing/ward/treatment team**	3	3	0
**Intranet**	3	0	0
**Screening**	3	0	0
**During the patient visit**	1	0	-
***Via*** **the weekly task force meeting**	1	1	0
**No special measures**	20	25	5

### Burden of the Psychosocial Teams, Needs, and Requests for Future Development of COVID-psyCare

**Figure 6:**
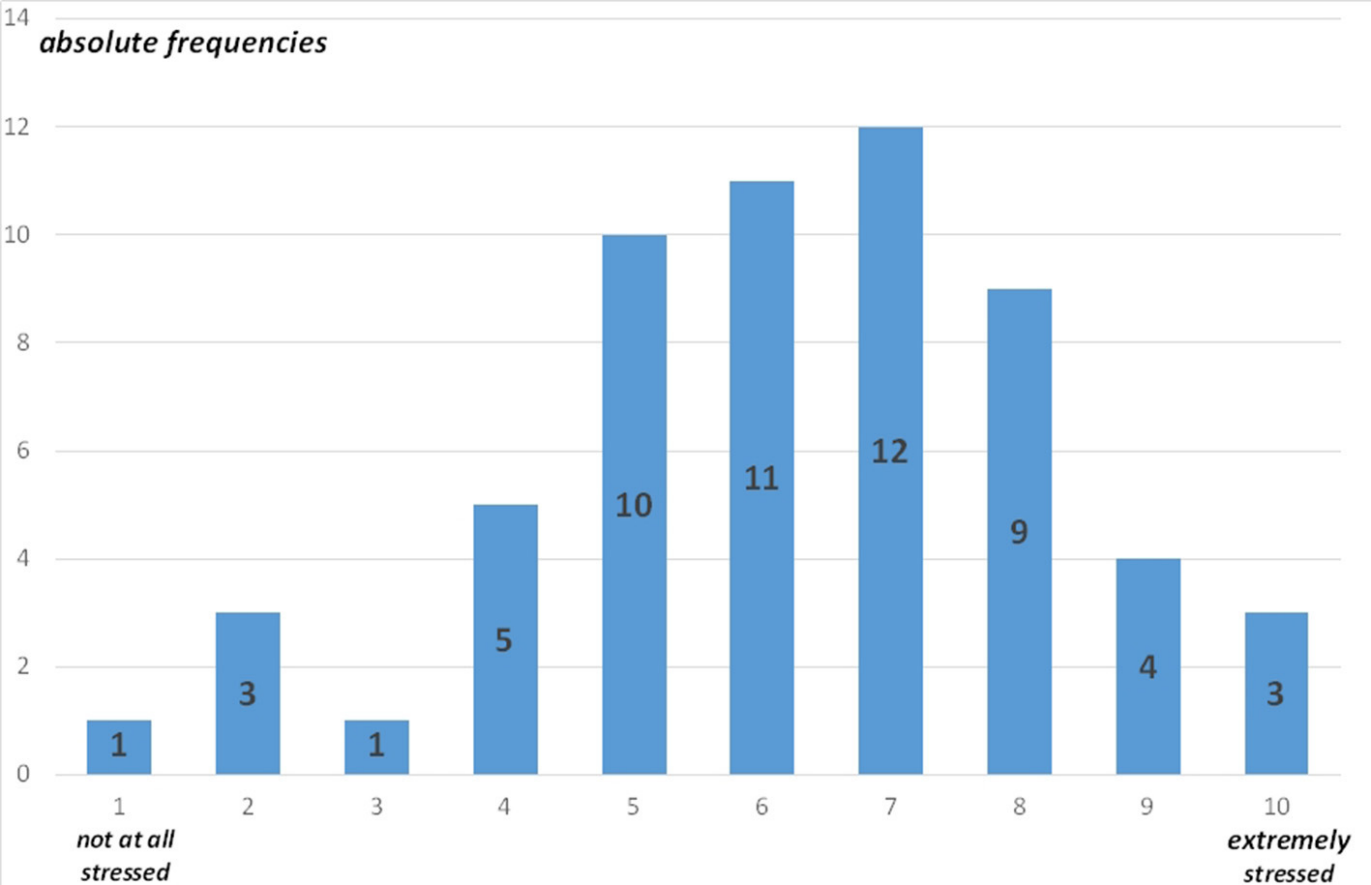
Maximum burden of the COVID-19 pandemic on the psychosocial teams (59 entries, absolute frequencies).

The maximum burden of the COVID-19 pandemic on the psychosocial teams (mean 6.24, 1–10, SD: 2.04) is depicted in Figure 6.

**Figure 7:**
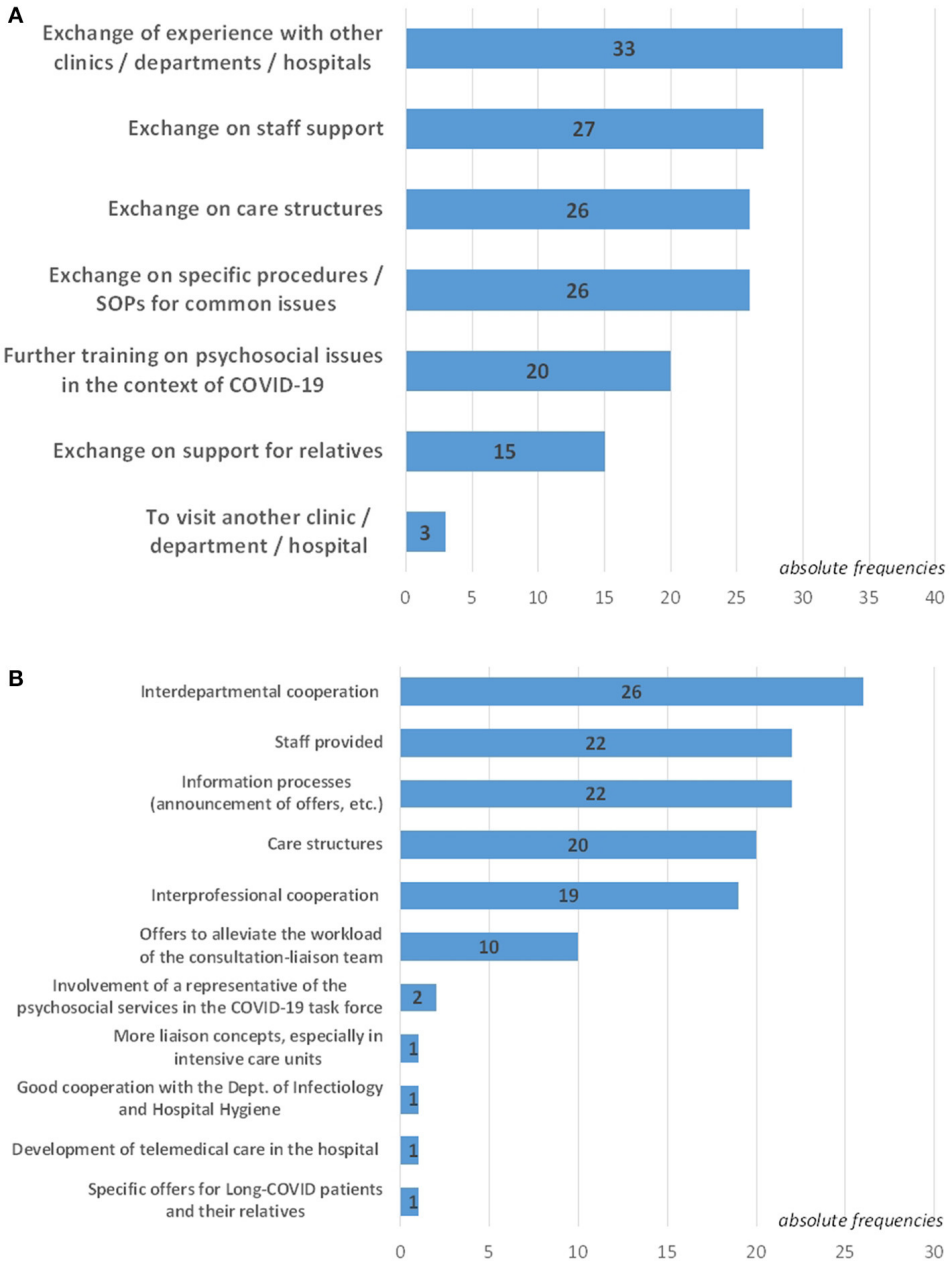
Needs and requests for the future. **(A)** Specification of required exchange/support regarding COVID-psyCare (*n* = 37). (**B)** Specification of changes/improvements that are considered essential for the future with regard to psychosocial care services in one's hospital in the COVID-19 context (*n* = 39).

Figure 7 displays the needs and requests for the future of COVID-psyCare. Among the 77 CL services that reported providing COVID-psyCare, 37 (48%) expressed requests for exchange/support (Figure 7A), 39 (51%) suggested changes/improvements that they considered essential for the future (Figure 7B).

### Additional COVID-19-Related Aspects With Relevance to CL Services

The following aspects were emphasized as additionally relevant to CL services in the context of the COVID-19 pandemic:

- *Regarding structures and general:* Clear structures and communication; clear allocation of responsibilities as well as spatial and time resources; technical equipment of workplaces with webcam and headset; establishment of intensified collaboration with pastoral care; sensitization to psychosocial emergency care.
- *Regarding staff:* Sufficient personal protection equipment (PPE) for the staff; update and training on COVID-specific hygiene measures and how to use PPE for medical and non-medical personnel early in the pandemic; sensitization to teams and employees; reducing the anxiety of the staff; clear regulations in cases of suspected COVID-19 infection (before and after the event); staff support also giving relevance to issues that have not been taken into account so far.
- *Regarding patients:* Problems with information technology (IT) for the often geriatric clientele; early involvement in the treatment of COVID-19 patients and their relatives, especially in ICUs.

## Discussion

### Key Results

So far, there is little data on psychosocial care by psychosomatic, psychiatric, and psychological CL services in general hospitals during the COVID-19 pandemic. Referring to our three study objectives, our online survey of such services in Germany, Austria, and Switzerland provided the following key results:

(1) Health care in the COVID-19 pandemic frequently seems to require the development of additional cooperation structures to foster good interdisciplinary and interprofessional cooperation.
(2) Among 77 CL services reporting COVID-psyCare, 26 (34%) offered specific interventions for patients, 19 (25%) for relatives, and 46 (60%) for staff. Overall, COVID-19 brought the psychosocial burden of the hospital staff more into focus. Nevertheless, regarding the time resources provided for COVID-psyCare, most of the time (61%) was used for the treatment and psychosocial support of COVID-19 patients, following the main focus of CL services on patient care.
(3) For optimized current and future pandemic response, there is a relevant demand for exchange between CL services and for improvements of psychosocial care services in general hospitals.

### Characterization of Participating Hospitals and CL Services

In terms of somatic involvement and maximum workload, we were predominantly dealing with hospitals involved in the somatic care of COVID-19 patients. The characteristics of the study respondents were very heterogeneous. The reported psychosocial care structures were diverse and colorful. Historically, hospitals appear to have developed a broad and heterogeneous landscape of psychosocial care structures. The different characteristics of psychosomatic, psychiatric, and psychological CL services in Germany, Austria, and Switzerland reflect the heterogeneity of psychosocial care in these countries.

### Provision of COVID-psyCare Among Participating CL Services

Up to the third wave of the COVID-19 pandemic, 79% of participating CL services in Germany, Austria, and Switzerland reported an additional provision of COVID-psyCare. Among these CL services, 61% reported that additional cooperation structures had been established within the hospital for COVID-psyCare. The partners involved in these cooperation structures are in descending order: Psychosomatic Medicine, Pastoral care, Psychiatry, Social services, the Nursing team, Palliative care, Psychology/Medical psychology, Psycho-oncology, Child and adolescent psychiatry, Clinical ethics, Human resources, Workplace health promotion, and Work psychology. These findings indicate that interdisciplinary and interprofessional work has been intensified in dealing with the COVID-19 pandemic. On the other hand, 21% of psychosomatic, psychiatric, and psychological CL services had not developed specific structures or procedures in the context of COVID-19.

### Target Groups of COVID-psyCare for CL Services

For a general comparison of the kind of psychosocial care regularly provided by psychosocial CL services older data from the European Consultation-Liaison Workgroup (ECLW) Collaborative Study are available (17). The final sample of CL services consisted of 56 services from 11 European countries, including 226 consultants seeing 14,717 patients during 1 year in consultation. The study reported a consultation rate of 1% (median; 1.4% mean). The consultant involved the patient's family in 16.3% of the cases. In 44.6% of the cases, the ward staff was also the focus of intervention, however primarily patient-related. In our study, reported interventions were targeted to the bio-psycho-social care of hospitalized COVID-19 patients and their relatives. Additional services and offers were developed to reduce the immense psychosocial burden of staff members in the hospital (18). The most common ways of communication to reach these target groups were in-person contacts, word-of-mouth recommendations, and the internet. In addition, staff members were frequently informed of COVID-psyCare services *via* senior executives.

Many CL services implemented telepsychiatry options for patients as well as for staff (16, 19–21). During the COVID-19 pandemic, it became evident that for many patients, telepsychiatry consultation works well (22), but problems with IT were met for the often geriatric clientele; all in all, a substantial portion of CL work must be performed face-to-face, and it is necessary to triage for appropriateness for telepsychiatry consultation (2).

### Specific Services of COVID-psyCare for Patients

The COVID-19 pandemic has been global health as well as economic shock. Regarding the general population, in a multicentric study (*n* = 20,712 participants) from Italy, one of the western countries most severely hit by COVID-19, access to mental health services during the pandemic was reported in 7.7% of cases (23); among those referred to mental health services, in 93.9% of the cases (*n* = 1,503 subjects), a psychological assessment was requested and in 15.7% of the cases (*n* = 252) a psychiatric consultation. In hospitalized COVID-19 patients, COVID-19 comes with a high incidence of psychological distress and neuropsychiatric symptoms; up to 85% of critically ill COVID-19 patients have neuropsychiatric manifestations; also uninfected patients are affected by the pandemic with psychosocial distress and worsening preexisting mental disorders (2, 24, 25). Confronted with this situation, 34% of CL services providing COVID-psyCare reported additional offers for patients. Overall, 61% of the time devoted to COVID-psyCare was directed to patients. Specific services of COVID-psyCare for patients reported in our survey are in descending order: Corona telephone hotline for patients, consultation staff specifically for corona referrals, specific procedures/standard operating procedures (SOPs) for common questions, e.g., for dealing with the anxiety of COVID-19 patients, psychosocial corona-care-team, liaison staff on COVID wards, aftercare services for patients with the post-COVID syndrome, a corona outpatient clinic, switch to telephone consultations - if reasonable and possible, and online services with information and psychosocial support. When asked to what extent these interventions have proven successful, the most favorably assessed and frequently applied interventions for patients were the provision of liaison staff on COVID wards and consultation staff for COVID-19 specific referrals, as well as the provision of specific protocols/SOPs for common questions, e.g., dealing with the anxiety of COVID-19 infected patients.

### Specific Services of COVID-psyCare for Relatives of COVID-19-Infected Patients

Illness-related, psychosocial, and hospital-related factors are risk factors for clinically relevant psychological distress in relatives of COVID-19-infected patients; resilience was negatively associated with anxiety, depression, and PTSD in relatives (1). In dealing with this challenge, 25% of CL services providing COVID-psyCare reported additional offers for relatives. Overall, 12% of the time devoted to COVID-psyCare was directed to relatives. Specific services of COVID-psyCare for relatives reported in our survey are in descending order: Corona telephone hotline for relatives, specific counseling for relatives, specific procedures/SOPs for supporting relatives, an information sheet for relatives, imparting and organization of internet-based video contact opportunities, organization and accompaniment of on-site visits, accompaniment after a death. Frequently offered procedures for relatives rated as most successful were the provision of specific protocols/SOPs for supporting relatives and specific counseling for relatives.

### Specific Services of COVID-psyCare for Hospital Staff

About 60% of CL services providing COVID-psyCare reported additional offers for health care personnel. On average, 27% of the time devoted to COVID-psyCare was directed to hospital staff, as estimated by study respondents. This is as per recent findings on the psychosocial burden of the medical staff, including increased depression/depressive symptoms, anxiety, psychological distress, and poor sleep quality (9, 24, 26–28). Therefore, it is critical that health care organizations have systems in place to support institutional and individual resilience (2). According to our study, CL services seem to be suitable structures to offer adequate support to staff members in the hospital in times of crises.

Specific services of COVID-psyCare for hospital staff reported in our survey are in descending order: Consultation hours/counseling for staff, a telephone hotline for staff, case discussions on patient-related stressful situations, team supervision/facilitated group exchange on how the corona situation was experienced, workshops to strengthen resilience, creating chill-out opportunities for high-stress teams, work with team leaders on helpful support measures for staff/teams, training in dealing with psychosocial stress of patients and relatives, recommendations for mental hygiene, preparations and discussions on how to deal with triage situations, ethical online consultations, debriefing/ daily review at the ICU, and spiritual support. Among these offers, team supervision and case discussions on patient-related stressful situations were most favorably assessed as having proven successful. In summary, especially interventions related to the liaison function of CL services seem to be perceived as highly useful. Additionally, a similar positive rating was also reported for specific protocols/SOPs that offer guidance for staff members to manage specific challenges in the provision of psychosocial care for COVID-19-infected patients and their relatives.

Ideally, an integrated continuum of care approach should be instituted, including E-Mental Health Interventions (21), crisis leadership consultation and training, staff peer support teams, multidisciplinary rounds, recreation spaces, wellness programs, support groups, and psychological/psychiatric services (29–31). Such coordinated programs may be cost-effective because of their positive effects on absenteeism and turnover (32, 33). A problem may be that hours devoted to staff support usually are non-billable. Of note, although such services have low rates of utilization - lower for medical doctors than for nurses - informal contact with CL staff may enhance interest in service use (2).

Based on the results of a large cross-sectional study from Germany, the COVID-19 pandemic has greatly impacted the following groups, work environments, and living situations: Women, employees with a migrant background, younger employees, individuals with private obligations to care for children and dependents, men concerning dysfunctional coping strategies, people living alone and, when compared to other occupational groups, frontline workers, such as nurses/paramedics and medical technicians (10). To lay the best basis for healthy and efficient work, it seems necessary to take measures especially tailored to the needs of these different groups of HCWs.

### Need for Further Exchange

Our findings point to a relevant demand for further exchange between psychosomatic, psychiatric, and psychological CL services on COVID-psyCare in general hospitals. Issues for this exchange expressed in our survey are in descending order: Exchange of experience with other clinics/departments/hospitals, exchange on staff support, on care structures, on specific procedures/SOPs for common issues, further training on psychosocial issues in the context of COVID-19, and support for relatives, finally, to visit another clinic/department/hospital, e.g., for an exchange on experience with different SOPs.

### Requests for the Future

Many of the same concerns as for other HCWs can be expected to apply to CL professionals as well - fear of infection, fear of transmitting illness to others, traumatizing experiences during hospital work, moral injury, and burnout (2, 5, 34). In line with this assumption, this study reports a mean value of 6.24 (SD: 2.04) for the maximum burden of the COVID-19 pandemic on the psychosocial teams on a scale of 1 (“not stressed at all”) to 10 (“extremely stressed”). Several changes considered essential for the future concerning CL services in the context of COVID-19 were chosen in descending order: improvement of interdepartmental cooperation, the provision of more CL service staff, information processes (announcement of offers, etc.), care structures, interprofessional cooperation, offers to alleviate the workload of the CL team, involvement of a representative of the psychosocial services in the COVID-19 task force, more liaison concepts—especially in ICUs, good cooperation with the Department of Infectiology and Hospital Hygiene, further development of telemedical care in the hospital as well as specific offers for long COVID patients and their relatives.

### Limitations

A strength of our study is that it gives information from a naturalistic health services research project. However, this comes with several limitations: First, comparability between CL services on a national level is limited due to substantial heterogeneity of health care systems and organizational models of psychosomatic, psychiatric, and psychological CL services in Germany, Austria, and Switzerland. Second, a selection bias needs to be considered when interpreting the results of this study: As the study has been initiated by spokespersons of psychosomatic CL services, a lower threshold to participate in this study may have existed for psychosomatic as compared to psychiatric and psychological CL services despite structured efforts to reach as many psychiatric, psychosomatic, and psychological CL services as possible. Especially in Germany and Austria, where the professional societies for Psychiatry did not participate in the mailing of the study, psychiatric CL services were underrepresented. Furthermore, it is possible that mainly CL services that have established COVID-psyCare participated in this survey leading to an overestimation of its provision. Third, when interpreting the fact that ~79% of participating CL services have reported the provision of specific COVID-psyCare whereas approximately 21% did not provide such care, it needs to be considered that the reported level of involvement of the hospitals in the somatic care of COVID-19 patients also differs significantly. A total of 74 general hospitals/CL services (76%) reported medium to very strong involvement and 24 general hospitals/CL services (24%) reported no or low involvement in the somatic care of COVID-19 patients. Fourth, it became apparent that precise national denominators of CL services were not available and that it was very difficult to get a good estimate for the total number of CL services in the participating countries. Fifth, the response rate of our study was limited to 55% of the surveyed CL services. Overall, the CL services that responded to our survey may not fully represent the entire field; thus, the generalizability of our results is restricted. Sixth, we used an *ad hoc* developed questionnaire for the survey, which may further affect the generalizability of our findings. However, to the best of our knowledge, there are practically no referenceable instruments, as the COVID-19 pandemic represents an unprecedented public health emergency. Seventh, each response was finished by one representative of the respective CL service which presumably brought subjective bias to the response. Eighth, data quality was affected by missing answers. Finally, our survey period from 12/2020 to 05/2021 met a similar, yet, a somewhat different, second and third wave of COVID-19 infections in Germany, Austria, and Switzerland which may have influenced the study results (for a comparison of figures of waves of COVID-19 infection numbers and death rates during the survey period see, for example, https://coronavirus.jhu.edu/map.html) (8); however, the broad professionalization in dealing with this new challenge took place mainly at the beginning of the pandemic in spring 2020 of which we believe to be able to give an informative insight. Therefore, the main measures may have been taken before the time of the survey. Likewise, services might also have been further adapted after the end of this survey in May 2021.

### Clinical and Organizational Implications - Lessons Learned From This Survey

In the following, we summarize the lessons learned from this survey, concerning the clinical and organizational implications of our study. Thereby, our findings are put into perspective by referring to the viewpoints of international scientific associations such as the Academy of Consultation-Liaison Psychiatry (ACLP) (2), the European Psychiatric Association (EPA) (35), and the World Psychiatric Association (WPA) (36). Given the likely lagged effects of the COVID-19 pandemic on health and the economy, the total demand for mental health services is likely to stay at increased levels, potentially for several years (35). According to the main focus of CL services on patient care, most of the time resources provided for COVID-psyCare are needed for the treatment and psychosocial support of COVID-19 patients with and without mental disorders (36). Additionally, staff support is gaining in importance: Health and social care workers and other frontline professionals who have experienced high levels of psychological distress during the crisis require sustained support. Efforts to protect their general and mental health need to be scaled up (2, 35): To be better prepared for future challenges like pandemics or catastrophes measures supporting the maintenance of the health status of staff members should be implemented comprehensively, especially as preventive procedures (2). The staff should be involved in this process to create tailored measures; a combination of top-down and bottom-up approaches might be most successful. In the sense of precision medicine, CL offers should be more specifically tailored to vulnerable groups concerning gender, age, family and living situation, migrant background, frontline workers, etc. (10). A greater focus will be put on resilience. This survey underlines the importance of liaison concepts: Summarizing, particularly those interventions for patients, their relatives, and for hospital staff, that are typically associated with the liaison function of CL services seem to be perceived as highly beneficial, not only in times of crisis. This suggests a high level of implementation and integration of most participating CL services in their hospitals (11). However, still, liaison models often are not implemented because of restricted financial resources or a lack of awareness concerning the importance of liaison services. Hospital management and health policy could contribute to better care of patients and better support of HCWs by implementing liaison concepts. Within the health care system, the development of additional cooperation structures seems necessary to foster good interdisciplinary and interprofessional cooperation with the aim of more integrated mental health care that is better linked to primary and community services. Our findings also confirm that the pandemic has increased the provision of telemedicine services (2, 35). The task will be to reshape mental health service delivery in a way that traditional mental health service models can reasonably be blended with digital services. Of course, long-term mental health support plans need to be tailored to individual country contexts (35).

### Research Implications

Our study attracted great international interest indicating a great need for international exchange on psychosocial health care in the context of the COVID-19 pandemic. According to this need, our study has been expanded into an international survey in 11 other European countries, Iran, and parts of Canada (ClinicalTrials.gov NCT04753242). We will report on this large international survey and its results elsewhere. Considering that the survey questions involve issues of patients, their relatives, and staff in general hospitals, future studies should make efforts to collect data from them to obtain more comprehensive results. Further studies should analyze the prevalence of COVID-19 infection, morbidity, mortality, and mental health of CL professionals during the pandemic (2).

## Conclusion

In summary, the COVID-19 pandemic seems to have put things under a burning glass and has highlighted problems in our health care systems. The results of our survey underline the crucial role of psychosomatic, psychiatric, and psychological CL services in an integrative and comprehensive health care approach to the challenges of the COVID-19 pandemic in general hospitals. They illustrate reported adjustments of CL service structures to meet the most urgent challenges of this pandemic in the somatic hospital setting by the provision of COVID-psyCare for patients, their relatives, and hospital personnel.

## Data Availability Statement

The raw data supporting the conclusions of this article will be made available by the authors, without undue reservation.

## Ethics Statement

Ethical clearance for this study was acquired from the Ethics Committee of Northwest and Central Switzerland in Basel/ Switzerland, the Ethics Committee of the Medical Faculty of the University of Cologne/ Germany, and the Ethics Committee of the Medical University of Graz/ Austria. Each participant provided informed consent that his/her responses could be used for analyses that would be reported in scientific publications.

## Author Contributions

RS, BS, CF, CH, FV, and GM contributed to the conception and design of the study. RS organized the translation of the survey and wrote the first draft of the manuscript. GM, RS, and FV ensured clarifications on data protection. RS, BS, CF, and FV took care of the necessary ethical votes. RS and CH arranged study registration. GM and NR programmed the online survey. RS, BS, CF, CH, FV, SS, and UH contributed to the dissemination of the survey. BS and FV were study contact persons for Germany, CF for Austria, CH and RS for Switzerland. NR, GM, and BS organized the database. BS performed the statistical analysis. CF and FV wrote sections of the manuscript. All authors contributed to the development of the survey questionnaire, interpretation of the data, manuscript revision, and read and approved the final manuscript.

## Conflict of Interest

The authors declare that the research was conducted in the absence of any commercial or financial relationships that could be construed as a potential conflict of interest.

## Publisher's Note

All claims expressed in this article are solely those of the authors and do not necessarily represent those of their affiliated organizations, or those of the publisher, the editors and the reviewers. Any product that may be evaluated in this article, or claim that may be made by its manufacturer, is not guaranteed or endorsed by the publisher.

## Supplementary Material

Data sheet 1 English version of the online survey.

Data sheet 2 German version of the online survey.

## Abbreviations

ACLP: Academy of Consultation-Liaison Psychiatry
CL services: Consultation and liaison services
COVID-19: Coronavirus disease 2019
COVID-psyCare: COVID-19 related psychosocial care
DGPM: German Society for Psychosomatic Medicine and Medical Psychotherapy
DKPM: German College of Psychosomatic Medicine
ECLW: European Consultation-Liaison Workgroug
EKNZ: Ethikkommission Nordwest- und Zentralschweiz (Ethics Committee of Northwest and Central Switzerland)
EPA: European Psychiatric Association
FTE: Full-time equivalent
HCW: Health care worker
ICU: Intensive care unit
IT: Information technology
ÖGPPM: Austrian Society for Psychosomatics and Psychotherapeutic Medicine
PPE: Personal protection equipment
PTSD: Posttraumatic stress disorder
SAPPM: Swiss Academy of Psychosomatic and Psychosocial Medicine
SSCLPP: Swiss Society of Consultation-Liaison Psychiatry and Psychosomatics
SD: Standard deviation
SOP: Standard operating procedure
WPA: World Psychiatric Association
95% CI: 95% Confidence interval.
