# Supplementary material for: COVID-19-Related Psychosocial Care in General Hospitals: Results of an Online Survey of Psychosomatic, Psychiatric, and Psychological Consultation and Liaison Services in Germany, Austria, and Switzerland
Source: Front Psychiatry. 2022 Jun 24;13:870984. doi: 10.3389/fpsyt.2022.870984 (PMC9270003; doi:10.3389/fpsyt.2022.870984)
Supplement: Data sheet 1 — English version of the online survey. [file Data_Sheet_1.PDF]

Online survey
of the Psychosomatic Consultation-Liaison Working Group
on dealing with the COVID-19 pandemic

Online survey

Welcome! Willkommen! Bienvenue! Benvenuti!

Please first select the language (English, German, French, Italian) at the top right of this page.

Bitte wählen Sie zuerst oben rechts auf dieser Seite die Sprache aus (Englisch, Deutsch, Französisch, Italienisch).

Veuillez d'abord sélectionner la langue en haut à droite de cette page (anglais, allemand, français, italien)

Si prega di selezionare la lingua in alto a destra in questa pagina (inglese, tedesco, francese, italiano).

Dear colleagues

The COVID-19 pandemic raised new challenges in supporting patients, relatives and staff for psychosomatic, psychiatric and psychological consultation and liaison (C&L) services in general hospitals. We want to build upon the experience gained so far in order to be well prepared for the future.

Therefore, we kindly ask you to participate in the survey.

The aims of this survey are
(1) To summarize the efforts made in psychosocial care in the context of the COVID-19 pandemic.
(2) To assess the experiences made with the established services so far.
(3) To assess the need for networking, cooperation and support.
(4) Improvement of psychosocial care in acute hospitals in the context of pandemics.

The survey is being led by the Psychosomatic Consultation-Liaison Working Group of the German College of Psychosomatic Medicine (DKPM) and the German Society for Psychosomatic Medicine and Medical Psychotherapy (DGPM) in cooperation with the Swiss Academy for Psychosomatic and Psychosocial Medicine (SAPPM), the Swiss Society for Consultant-Liaison Psychiatry and Psychosomatics (SSCLPP) and the Austrian Society for Psychosomatics and Psychotherapeutic Medicine (ÖGPPM).

Only one person per psychosomatic, psychiatric or psychological C&L service should take part, in agreement with the respective management.

Participation in this survey is possible without mentioning your name.
Registration is not required for participation.

Only pseudonymised data is collected and processed. Completing the questionnaire takes about 15 minutes. Your participation in the survey is voluntary. You can cancel the survey at any time and without giving reasons. This will not affect you adversely in any way. You also have the right to information and correction, the right to deletion, the right to restriction and the right to data portability (Art. 13 para. 2 lit. b) of the processed and not yet fully anonymised data, as well as the possibility to revoke your consent even after completion of the questionnaire. For this purpose, we ask you to generate an individual code at the beginning of the survey, which allows us to pseudonymise and thus identify the corresponding data set if necessary.
Data collection is carried out with the online survey tool Questback EFS Fall 2019 / licence model "Unipark" of Questback GmbH via the University of Basel. Questback stores the data collected via the tool in the server park in Frankfurt. This is reliably protected from external access. The BSI-certified data centre is subject to extremely high data protection and security requirements according to ISO 27001 based on IT-Grundschutz. Subsequently, the data is stored, processed and statistically evaluated at the University of Basel and the University Hospital Basel. Further processing of the data and interpretation of the results is carried out in cooperation with German, Austrian and Swiss members of the above-mentioned working groups and societies. For this purpose, completely anonymised data sets will be passed on to the participating members of the working groups and societies if necessary.
After completion of the study, all data will be stored and archived on a secure server of the University of Basel or the University Hospital Basel in accordance with applicable law (in particular the European Data Protection Regulation, DSGVO, and Swiss data protection law). The duration of data retention is 10 years.
The personal data (individual code; perspective from which you answer the survey) collected in this study are kept to a minimum.
In the context of this study, publications are planned by the various institutions involved. In case of publication of study results, the confidentiality of your data remains guaranteed.

To start the survey, please confirm your consent and the related clarification below.

If you have any questions or comments about this study or would like to be informed about the research results, please feel free to contact us.

Thank you very much for your participation and kind regards.

· Prof. Dr. med. Rainer Schäfert, Basel
· Dr. phil. Dipl.-Psych. Barbara Stein, Nürnberg
· Dr. med. Frank Vitinius, Köln
· PD Dr. med. Christian Fazekas, Graz

Contact details:
- Prof. Dr. med. Rainer Schäfert
University Hospital Basel, Clinic for Psychosomatics, Hebelstrasse 2, CH-4031 Basel, Tel.: +41 61 265 52 94, eMail: covid-survey-medizin@unibas.ch
- Dr. phil. Dipl.-Psych. Barbara Stein
Nuremberg Hospital, Paracelsus Medical Private University Nuremberg, Clinic for Psychosomatic Medicine and Psychotherapy,
Prof. Ernst-Nathan-Str. 1, 08.U1.118, D-90419 Nuremberg, Tel.: +49 911 398-113673 or -2839, eMail: barbara.stein@klinikum-nuernberg.de
- Frank Vitinius, MD
University Hospital Cologne, Clinic and Polyclinic for Psychosomatics and Psychotherapy, Kerpener Str. 62, D-50937 Köln (Cologne), Tel.: +49 221 478 98649, eMail: frank.vitinius@uk-koeln.de
- PD Dr. med. Christian Fazekas
Medical University of Graz - University Hospital Graz, University Department of Medical Psychology and Psychotherapy, Auenbruggerplatz 3, A-8036 Graz, Tel.: +43 316 385 12516, eMail: christian.fazekas@medunigraz.at

.

I hereby give my consent that the answers submitted by me within the framework of the study and relating to my institution may be stored and evaluated at the University of Basel and the University Hospital Basel in pseudonymised form, passed on in anonymised form to the other participating institutions in Germany and Austria and used to answer scientific questions. Furthermore, I agree that the data may be used in anonymised form for scientific presentations and publications. I acknowledge that I can cancel my participation at any time without giving reasons.

☐ I have been informed that I can terminate my participation in the study at any time. This also includes the right to revoke my consent to data processing. In this case, the personal reference to the data will be deleted.

The following ethics committees were consulted and the necessary ethics approvals or declarations of non-responsibility were obtained in accordance with the legal requirements: vote of the ethics committees of the Medical Faculty of the University of Cologne (20-1416), declaration of the Ethics Committee Northwestern and Central Switzerland (EKNZ) (Req-2020-00861).

I have been informed that the data collected will be stored, passed on and analysed in pseudonymised form. The pseudonymisation process takes place by asking the respondent to generate a personal code instead of their full name. This code serves

as a key that can only be reproduced by the respondent, but not - without disproportionate effort - by the study management or by third parties.

- You are not recognisable as a person in the evaluation. Furthermore, you are granted the right to information and correction of incorrectly processed data. You have the right to inspect your data that is collected during the study. If you discover errors in your data, you have the right to have them corrected by Prof. Dr. med. Rainer Schäfert. If you have any questions about the study, you can contact the persons listed below.
- For participants from the European Union: I have been informed that my data will be transferred to a third country - in this case Switzerland - outside the EU by entering the data directly on the survey platform and the server is located in Switzerland (cf. Art. 13 Para. 1 lit. c and f DSGVO). The European Commission has established an adequate legal level of data protection in Switzerland (so-called adequacy decision). You can find this adequacy decision at https://edps.europa.eu/data-protection/data-protection/glossary/a_de.

- We are generally obliged to provide you with a copy of your personal data free of charge. (Art. 15 para. 3 DSGVO).
- You have the right to contact the responsible data protection officers:

- University Hospital Basel: Danielle Kaufmann; eMail: danielle.kaufmann@unibas.ch or Datenschutzbeauftragte@usb.ch.
- Medical University of Graz: Univ.-Prof. Dr. Thomas Wagner; eMail: datenschutz@medunigraz.at
- University of Cologne: 1st Data Protection Officer of the University of Cologne: eMail: dsb@verw.uni-koeln.de; 2nd Data Protection Officer of the University Hospital Cologne: datenschutz@uk-koeln.de.

You also have the right to lodge a complaint with the competent supervisory authority for data protection: The State Commissioner for Data Protection of North Rhine-Westphalia, Kavalleriestrasse 2-4, 40213 Düsseldorf, Germany.

**1.**	We would like to make it possible for your details to be subsequently deleted at your request, but without asking you to give your name for this purpose. Therefore, we ask you to generate a code that only you know and that we can use to identify your record if necessary. The code consists of the following elements:
	1. First letter of the mother's first name<br>2. First letter of the father's first name<br>3. Day of birth of the mother in two digits (if 15.03.1948, then 15)<br>4. Year of birth of the mother in two digits (if 1948, then 48).<br><br>My code: _______________

2.	Questions about the hospital and department to which this survey refers:
2.a	**Which hospital do your responses refer to?**
	1 Name of hospital (free text): ....<br>………………………………………………………………………………………….<br><br>2 Department (free text): ....<br>…………………………………………………………………………………………
2.b	**In which city is your hospital (mainly) located?**
	(free text):<br>…………………………………….……………………………………………………..

3.	In which state and federal state or canton is your hospital located?	
	1 ☐ Deutschland	1.1 ☐ Baden-Württemberg<br>1.2 ☐ Bavaria<br>1.3 ☐ Berlin<br>1.4 ☐ Brandenburg<br>1.5 ☐ Bremen<br>1.6 ☐ Hamburg<br>1.7 ☐ Hesse<br>1.8 ☐ Mecklenburg-Western Pomerania<br>1.9 ☐ Lower Saxony<br>1.10 ☐ North Rhine-Westphalia<br>1.11 ☐ Rhineland-Palatinate<br>1.12 ☐ Saarland<br>1.13 ☐ Saxony<br>1.14 ☐ Saxony-Anhalt<br>1.15 ☐ Schleswig-Holstein<br>1.16 ☐ Thuringia

	2 ☐ Austria	2.1 ☐ Vorarlberg<br>2.2 ☐ Tyrol<br>2.3 ☐ Salzburg<br>2.4 ☐ Carinthia<br>2.5 ☐ Styria<br>2.6 ☐ Upper Austria<br>2.7 ☐ Lower Austria<br>2.8 ☐ Burgenland<br>2.9 ☐ Vienna
	3 ☐ Schweiz	2.1 ☐ Aargau<br>2.2 ☐ Appenzell Innerrhoden<br>2.3 ☐ Appenzell Ausserrhoden<br>2.4 ☐ Berne<br>2.5 ☐ Basel Country<br>2.6 ☐ Basel City<br>2.7 ☐ Fribourg<br>2.8 ☐ Geneva<br>2.9 ☐ Glarus<br>2.10 ☐ Grisons<br>2.11 ☐ Jura<br>2.12 ☐ Lucerne<br>2.13 ☐ Neuchâtel<br>2.14 ☐ Nidwalden<br>2.15 ☐ Obwalden<br>2.16 ☐ St.Gallen<br>2.17 ☐ Schaffhausen<br>2.18 ☐ Solothurn<br>2.19 ☐ Schwyz<br>2.20 ☐ Thurgau<br>2.21 ☐ Ticino<br>2.22 ☐ Uri<br>2.23 ☐ Vaud<br>2.24 ☐ Valais<br>2.25 ☐ Zug<br>2.26 ☐ Zurich

4.	**What type of hospital do you work in?**<br>*Please select only one of the following answers (single choice):*
	1 ☐ University hospital<br>2 ☐ General hospital<br>3 ☐ Specialized hospital<br>4 ☐ Attending hospital<br>5 ☐ Other type of hospital, if yes, which (free text):<br>……………………………………………….…

5.	**Which type of ownership does your hospital have?**<br>*Please select only one of the following answers (single choice).*
	1 ☐ Private ownership<br>2 ☐ Non-profit ownership (e.g. church organisation)<br>3 ☐ Public providers (incl. institutions under public law / public limited companies etc.)<br>4 ☐ Other ownership

6.	**How many beds does your hospital have in total?**
	_______ (*state number*)

7.	**From which professional perspective are you answering this survey?**<br>*(multiple answers possible)*

	[1] ☐ Psychosomatic Medicine<br>[2] ☐ Psychiatry<br>[3] ☐ Child and adolescent psychiatry and psychosomatics<br>[4] ☐ Medical psychology<br>[5] ☐ Psychological service / Psychological department<br>[6] ☐ other, if yes, which *(free text)*:<br>…………………………………………………………………………………………..

**8.**	**Which psychosocial services are available in your hospital?**<br>*(multiple answers possible)*
	[1] ☐ Psychosomatic consultation & liaison service<br>[2] ☐ Psychiatric consultation & liaison service<br>[3] ☐ Psychological consultation & liaison service<br>[4] ☐ other, if yes, which *(free text)*:<br>……………………………………………………………………………..…<br>…………………………………………………………………………..………

**9.**	**How many full-time equivalents does the consultation-liaison service of your own department have?**<br>*(Please answer in the form of a number in each case)*
	[1] ☐ Physician positions: ____<br>[2] ☐ Psychologist positions: ____<br>[3] ☐ Social worker positions: ____<br>[4] ☐ Nursing positions: ____<br>[5] ☐ Other full-time equivalents: ____

**10.**	**Involvement of your hospital in the somatic care of COVID-19 patients since the beginning of the pandemic.**
**10.a**	**What has been/is the maximum level of involvement of your hospital in the *somatic care* of COVID-19 patients since the beginning of the pandemic?**
	**0 – 1 – 2 – 3 – 4 – 5**<br>**(0 = not at all to 5 = very strongly)**
**10.b**	**To what extent was/is your hospital maximally occupied regarding the *somatic care* of COVID-19 patients?**
	**0 – 1 – 2 – 3 – 4 – 5**<br>**(0 = not at all to 5 = very strongly)**

**11.**	**Where were/are COVID-19 patients treated as inpatients in your hospital?**<br>*(multiple answers possible)*
	1 ☐ specially designated ward(s) for suspected cases<br>2 ☐ specifically designated ward(s) for COVID-19 patients<br>3 ☐ specially designated intensive care unit(s)/beds<br>4 ☐ other

**12.**	**Have other special structures for *somatic care* related to COVID-19 been established in your hospital?**
	1 ☐ no<br>2 ☐ yes and specifically *(multiple answers possible)*

	[2.1] ☐ Additional places for intensive care treatment
	[2.2] ☐ Additional places for artificial ventilation
	[2.3] ☐ Transformation of regular wards/areas for the treatment of COVID-19 patients
	[2.4] ☐ Transformation of regular wards/areas for the treatment of suspected COVID-19 cases
	[2.5] ☐ Outpatient assessment / testing centre for SARS-CoV-2/COVID-19
	[2.6] ☐ Expansion of specific laboratory capacities for testing for SARS-CoV-2

**13.a**	**Has any COVID-19 related psychosocial care been provided at your facility?**
	[1] ☐ No COVID-19 related psychosocial care has been provided at our facility.<br>[2] ☐ COVID-19 related psychosocial care has been provided at our facility.
**13.b**	**If at your facility COVID-19-related psychosocial care has been/is provided, how is the time spent on this care by the C&L mental health team in your hospital/department divided among the three target groups during the busiest times, roughly estimated in percentage terms?**<br>*(should add up to 100%)*
	Patients: _____%<br>Relatives: _____%<br>Staff members: _____%<br>**Total: 100 %**

14.	**Have cooperation structures been established within the hospital for psychosocial support in the context of the COVID-19 pandemic?**
	1 ☐ no<br>2 ☐ yes - if yes, who was involved? *(multiple answers possible)*
	3 ☐ Psychosomatic Medicine<br>4 ☐ Psychiatry<br>5 ☐ Palliative care<br>6 ☐ Psychology/medical psychology<br>7 ☐ Psycho-oncology<br>8 ☐ Pastoral care<br>9 ☐ Social services<br>10 ☐ Nursing team<br>11 ☐ other *(free text)*:<br>……………….……………………………..…………………………...…………

15.	**What structures for psychosocial care in the corona pandemic were developed in your hospital?**<br>*(multiple answers possible)*
	1 ☐ New care structures have been established.<br>2 ☐ Existing care structures were further developed.<br>3 ☐ The care structure remained unchanged.<br>4 ☐ No psychosocial care was/is possible
	Comment *(optional)*:<br>………………………………………………………………………………

16.	**Has a specific representative been appointed for psychosocial issues in the pandemic task force of your hospital?**

	[1] ☐ no<br>[2] ☐ yes and specifically: *(multiple answers possible)*
	[3] Internal person: Designation of a responsible person from the psychosocial departments<br>☐ Internal person: regular participation in meetings of the pandemic task force<br>☐ Internal person: selective participation in meetings of the pandemic task force
	[4] External person: Designation of a responsible person outside the psychosocial departments<br>☐ External person: regular participation in meetings of the pandemic task force<br>☐ External person: selective participation in meetings of the pandemic task force
	Comment *(optional)*:<br>…………………………………………………………………………………

**17.**	**What was/is the <u>maximum</u> availability of psychosocial care for COVID-19 patients in terms of time?**<br>*(multiple answers possible)*
	[1] ☐ daily for 24 hours<br>[2] ☐ daily, from 8 a.m. to midnight<br>[3] ☐ daily, during regular working hours, e.g. 8 a.m. to 5 p.m.<br>[4] ☐ Monday to Friday, 24 hours a day<br>[5] ☐ Monday to Friday, during regular working hours, e.g. 8 a.m. to 5 p.m.<br>[6] ☐ less than 5 selected days per week<br>[7] ☐ In addition to the above hours, there was on-call or stand-by duty.<br>[8] ☐ other – how? *(free text)*:<br>……………………………………………………………………………………………<br>……………………………………………………………………………………………

18.	**Have specific offers or procedures of psychosocial support <u>for patients</u> been established in your hospital in the context of COVID-19?**	**To what extent have these procedures / offers proven successful? 0 – 1 – 2 – 3 – 4 – 5 (0 = not at all to 5 = very strongly**
	[1] ☐ no<br>[2] ☐ yes – If yes, which ones? *(Multiple answers possible)*	
	[3] ☐ Specific procedures / *Standard Operation Procedures (SOPs)* for common questions	**0 – 1 – 2 – 3 – 4 – 5**
	[4] ☐ Consultation staff specifically for corona referrals	**0 – 1 – 2 – 3 – 4 – 5**
	[5] ☐ Liaison staff on COVID wards	**0 – 1 – 2 – 3 – 4 – 5**
	[6] ☐ Psychosocial Corona-Care-Team	**0 – 1 – 2 – 3 – 4 – 5**
	[7] ☐ Corona telephone hotline	**0 – 1 – 2 – 3 – 4 – 5**
	[8] ☐ Corona outpatient clinic	**0 – 1 – 2 – 3 – 4 – 5**
	[9] ☐ Aftercare services for patients with post-COVID syndrome	**0 – 1 – 2 – 3 – 4 – 5**

	[10] ☐ other – what? *(free text)*: ………………………………………………………………………… ………………………………………………………………………… ………………………………………………………………………… ………………………………………………………………………… ……………………………………………………………….………	**0 – 1 – 2 – 3 – 4 – 5**

**19.**	**Have specific offers or procedures of psychosocial support <u>for relatives</u> been established in the COVID-19 context in your hospital?**	**To what extent have these procedures / offers proven successful? 0 – 1 – 2 – 3 – 4 – 5 (0 = not at all to 5 = very strongly**
	[1] ☐ no<br>[2] ☐ yes – If yes, which ones? *(multiple answers possible)*	
	[3] ☐ Specific procedures / Standard Operation Procedures (SOPs) for supporting relatives	**0 – 1 – 2 – 3 – 4 – 5**
	[4] ☐ Corona telephone hotline also for relatives	**0 – 1 – 2 – 3 – 4 – 5**
	[5] ☐ Specific counselling offer for relatives	**0 – 1 – 2 – 3 – 4 – 5**
	[6] ☐ other – what? *(free text):* ……………………………………………………………………… ……………………………………………………………………… ……………………………………………………………………….. ……………………………………………………………………… ……………………………………………………………………….	**0 – 1 – 2 – 3 – 4 – 5**

**20.**	**Have specific offers or procedures of psychosocial support <u>for staff</u> been established in the COVID-19 context in your hospital?**	**To what extent have these procedures / offers proven successful? 0 – 1 – 2 – 3 – 4 – 5 (0 = not at all to 5 = very strongly**
	[1] ☐ no<br>[2] ☐ yes – If yes, which ones? *(multiple answers possible)*	
	[3] ☐ Case discussions on patient-related stressful situations	**0 – 1 – 2 – 3 – 4 – 5**
	[4] ☐ Team supervision / facilitated group exchange on how the corona situation is experienced as staff and in the team	**0 – 1 – 2 – 3 – 4 – 5**
	[5] ☐ Consultation hours/counselling for staff	**0 – 1 – 2 – 3 – 4 – 5**
	[6] ☐ Telephone hotline for staff	**0 – 1 – 2 – 3 – 4 – 5**
	[7] ☐ Targeted work with team leaders/supervisors on helpful support measures for staff/teams	**0 – 1 – 2 – 3 – 4 – 5**
	[8] ☐ Training in dealing with psychosocial stress of patients and relatives (recognition, communication, management)	**0 – 1 – 2 – 3 – 4 – 5**
	[9] ☐ Workshops to strengthen the resilience of staff (e.g. self-care / resource activation, "mental hygiene")	**0 – 1 – 2 – 3 – 4 – 5**
	[10] ☐ Creating a chill-out opportunities for high-stress teams	**0 – 1 – 2 – 3 – 4 – 5**

	11 ☐ other – what? *(free text):*<br>……………………………………………………………………………<br>……………………………………………………………………………<br>………………….…………………………………………………………<br>……………………………………………………………………………<br>…………………………………..……………………………………	**0 – 1 – 2 – 3 – 4 – 5**

**21.**	**Communication: How were/are the different target groups informed about the COVID-19 related psychosocial support services and offers?**<br>*(multiple answers possible)*
**21.a**	**Patients:**
	1 ☐ Flyer<br>2 ☐ Notice board<br>3 ☐ Internet<br>4 ☐ In person<br>5 ☐ Screening<br>6 ☐ Word-of-mouth recommendation<br>7 ☐ No special measures<br>8 ☐ Other, if yes, which *(free text)*:<br>……………………………………………………..…………………………...
**21.b**	**Relatives:**
	1 ☐ Flyer<br>2 ☐ Notice board<br>3 ☐ Internet<br>4 ☐ In person<br>5 ☐ Word-of-mouth recommendation<br>6 ☐ No special measures<br>7 ☐ other, if yes, which *(free text)*:<br>…………………………………………………………………………………...
**21.c**	**Staff:**

	[1] ☐ Flyer<br>[2] ☐ Notice board<br>[3] ☐ Internet<br>[4] ☐ In person<br>[5] ☐ Word-of-mouth recommendation<br>[6] ☐ Information via senior executives<br>[7] ☐ No special measures<br>[8] ☐ Other, if yes, which *(free text)*:<br>…..………………………………………………………………………………...

**22.**	**How would you rate the maximum burden of the COVID-19 pandemic on your own psychosocial team in your clinic / department?**		
**22.a)**	**on a scale 0 - 10 (0 = not at all stressed to 10 = extremely stressed)**		
	**not at all stressed**	**0 ———————————————10**	**extremely stressed**
**22.b)**	**During which period was the burden highest?**<br>*Please indicate the period in the following format: Ex. 01/2001*		
	**from (month/year)**	**to (month/year)**	

Needs and wishes for the future:

**23.**	**Do you or your team have any wishes for exchange/support regarding psychosocial care in the COVID 19 context?**
	[1] ☐ no<br>[2] ☐ yes – If yes, which ones? *(multiple answers possible)*

	3 ☐ Exchange of experience with other clinics / departments / hospitals<br>4 ☐ Exchange on specific procedures / Standard Operation Procedures (SOPs) for common issues<br>5 ☐ Exchange on care structures<br>6 ☐ Exchange on staff support<br>7 ☐ Exchange on support for relatives<br>8 ☐ To visit another clinic / department / hospital<br>9 ☐ Further training on psychosocial issues in the context of COVID-19<br>10 ☐ other – If yes, which? *(free text)*:<br>……………………………………………………………………………<br>……………………………………………………………………………<br>……………………………………………………………………………

**24.**	**Are there any changes / improvements that you consider essential for the future with regard to psychosocial care services in your hospital in the COVID-19 context?**
	1 ☐ no<br>2 ☐ yes – If yes, which ones? *(multiple answers possible)*
	3 ☐ with regard to care structures<br>4 ☐ with regard to interdepartmental cooperation<br>5 ☐ with regard to interprofessional cooperation<br>6 ☐ with regard to the staff provided<br>7 ☐ with regard to information processes (announcement of offers, etc.)<br>8 ☐ with regard to offers to alleviate the workload of the consultation-liaison team<br>9 ☐ other – if yes, what? *(free text)*:<br>…………………………………………<br>……………………………………………………………………………<br>……………………………………………………………………………

**25.**	**From your point of view, are there any other aspects in the COVID-19 context that are relevant to the consultation-liaison service and that you would like to add?**
	1 ☐ no<br>2 ☐ yes
	If yes, which ones? *(free text)*:<br>……………………………………………………………………………………<br>……………………………………………………………………………………<br>……………………………………………………………………………………

Thank you very much for your participation in this online survey!
In case of any questions, please do not hesitate to contact us:
eMail: covid-survey-medizin@unibas.ch
