# Supplementary material for: COVID-19-Related Psychosocial Care in General Hospitals: Results of an Online Survey of Psychosomatic, Psychiatric, and Psychological Consultation and Liaison Services in Germany, Austria, and Switzerland
Source: Front Psychiatry. 2022 Jun 24;13:870984. doi: 10.3389/fpsyt.2022.870984 (PMC9270003; doi:10.3389/fpsyt.2022.870984)
Supplement: Data sheet 2 — German version of the online survey. [file Data_Sheet_2.PDF]

Online-Umfrage der Arbeitsgruppe Konsiliar-Liaison-Psychosomatik zum Umgang mit der COVID-19 Pandemie

Online-Umfrage

Version 19, 27.11.2020

Willkommen! Bienvenue! Benvenuti!

Bitte wählen Sie zuerst oben rechts auf dieser Seite die Sprache aus (Deutsch, Französisch, Italienisch).

Veuillez d'abord sélectionner la langue en haut à droite de cette page (allemand, français, italien)

Si prega di selezionare la lingua in alto a destra in questa pagina (tedesco, francese, italiano).

Sehr geehrte Kolleginnen und Kollegen

Die COVID-19-Pandemie stellte Psychosomatische, Psychiatrische und Psychologische Konsiliar- und Liaison (K&L)-Dienste in Krankenhäusern der Akutversorgung vor neue Herausforderungen in der Unterstützung von Patient*innen, Angehörigen und Mitarbeitenden. Wir wollen die bislang gemachten Erfahrungen für eine Bilanzierung nutzen, um für die Zukunft gut gerüstet zu sein.

Daher bitten wir Sie herzlich um Ihre Teilnahme an der Befragung.

Ziele dieser Umfrage sind
(1) Bestandsaufnahme zur psychosozialen Versorgung im Kontext der COVID-19 Pandemie
(2) Bilanzierung der bislang mit den etablierten Angeboten gemachten Erfahrungen
(3) Erhebung des Bedarfs von Vernetzung, Kooperation und Unterstützung
(4) Verbesserung der psychosozialen Versorgung im Akutkrankenhaus im Kontext von Pandemien

Die Befragung wird federführend von der Arbeitsgruppe Konsiliar-Liaison-Psychosomatik des Deutschen Kollegiums für Psychosomatische Medizin (DKPM) und der Deutschen Gesellschaft für Psychosomatische Medizin und Ärztliche Psychotherapie (DGPM) in

Zusammenarbeit mit der Schweizerischen Akademie für Psychosomatische und Psychosoziale Medizin (SAPPM), der Schweizerischen Gesellschaft für Konsiliar-Liaisonpsychiatrie und -Psychosomatik (SSCLPP) und der Österreichischen Gesellschaft für Psychosomatik und Psychotherapeutische Medizin (ÖGPPM) durchgeführt.

Teilnehmen soll pro Psychosomatischen, Psychiatrischen oder Psychologischen K&L-Dienst nur jeweils eine Person in Abstimmung mit der jeweiligen Leitung.

Die Teilnahme an dieser Umfrage ist ohne die Nennung Ihres Namens möglich.
Eine Registrierung ist für die Teilnahme nicht erforderlich.

Die Erhebung und wissenschaftliche Verarbeitung der Daten erfolgt pseudonymisiert. Das Ausfüllen des Fragebogens dauert ca. 15 Minuten. Ihre Teilnahme sowie alle Angaben in der Befragung sind freiwillig. Sie können die Befragung jederzeit und ohne Angabe von Gründen abbrechen. Dies wirkt sich auf Sie in keiner Weise nachteilig aus. Ebenso haben Sie das Recht auf Auskunft und Berichtigung, das Recht auf Löschung, das Recht auf Einschränkung und das Recht auf Datenübertragbarkeit (Art. 13 Abs. 2 lit. b) der bearbeiteten und noch nicht vollständig anonymisierten Daten sowie die Möglichkeit Ihre Einwilligung auch nach Abschluss des Fragebogens zu widerrufen. Zu diesem Zwecke bitten wir Sie, zu Beginn der Umfrage, einen individuellen Code zu generieren, der uns eine Pseudonymisierung und damit die Identifikation des entsprechenden Datensatzes bei Bedarf ermöglicht.
Die Datenerhebung erfolgt mit dem Online-Befragungs-Tool Questback EFS Fall 2019 / Lizenzmodell „Unipark" der Firma Questback GmbH über die Universität Basel. Questback speichert die über das Tool gesammelten Daten im Serverpark in Frankfurt. Dieser ist zuverlässig vor externen Zugriffen geschützt. Das BSI-zertifizierte Rechenzentrum unterliegt extrem hohen Datenschutz- und Sicherheitsanforderungen nach ISO 27001 auf Basis des IT-Grundschutzes. Anschliessend werden die Daten an der Universität Basel und am Universitätsspital Basel gespeichert, aufbereitet und statistisch ausgewertet. Die weitere Verarbeitung der Daten und die Interpretation der Resultate erfolgt in Zusammenarbeit mit deutschen, österreichischen und schweizerischen Mitgliedern der oben genannten Arbeitsgruppen und Gesellschaften. Zu diesem Zwecke werden bei Bedarf vollständig anonymisierte Datensätze an die beteiligten Mitglieder der Arbeitsgruppen und Gesellschaften weitergegeben.
Nach Beendigung der Studie werden alle Daten entsprechend dem geltenden Recht (insbesondere der europäischen Datenschutzgrundverordnung, DSGVO, und dem schweizerischen Datenschutzrecht) auf einem sicheren Server der Universität Basel bzw. des Universitätsspitals Basel gespeichert und archiviert. Die Dauer der Datenaufbewahrung beträgt 10 Jahre.
Die personenbezogenen Daten (individueller Code; Perspektive, aus der Sie die Befragung beantworten), welche in dieser Studie erhoben werden, beschränken sich auf ein Minimum.
Im Rahmen dieser Studie sind Veröffentlichungen durch die verschiedenen beteiligten Institutionen geplant. Im Falle der Veröffentlichung von Studienergebnissen bleibt die Vertraulichkeit Ihrer Daten gewährleistet.

Um die Umfrage zu starten, bestätigen Sie bitte unten Ihr Einverständnis und die zugehörige Aufklärung.

Wenn Sie Fragen oder Bemerkungen zu dieser Studie haben oder über die Forschungsresultate informiert werden möchten, können Sie sich gerne bei uns melden.

Herzlichen Dank für Ihre Teilnahme und freundliche Grüsse

PD Dr. med. Christian Fazekas, Graz
Prof. Dr. med. Rainer Schäfert, Basel
Dr. phil. Dipl.-Psych. Barbara Stein, Nürnberg
Dr. med. Frank Vitinius, Köln

Kontaktdaten der Ansprechpartner:

- Prof. Dr. med. Rainer Schäfert
Universitätsspital Basel, Klinik für Psychosomatik, Hebelstrasse 2, CH-4031 Basel, Tel.: +41 61 265 52 94, eMail: covid-survey-medizin@unibas.ch
- Dr. med. Frank Vitinius
Universitätsklinikum Köln, Klinik und Poliklinik für Psychosomatik und Psychotherapie, Kerpener Str. 62, D-50937 Köln (Cologne), Tel.: +49 221 478 98649, eMail: frank.vitinius@uk-koeln.de
- Dr. phil. Dipl.-Psych. Barbara Stein
Klinikum Nürnberg, Paracelsus Medizinische Privatuniversität Nürnberg, Klinik für Psychosomatische Medizin und Psychotherapie, Dr. phil. Dipl. Psych. Barbara Stein
Prof. Ernst-Nathan-Str. 1 Haus 8, Zi. 08.U1.118, D-90419 Nürnberg, Tel.: +49 911 398-113673 oder -2839, eMail: barbara.stein@klinikum-nuernberg.de
- PD Dr. med. Christian Fazekas
Medizinische Universität Graz – Universitätsklinikum Graz, Universitätsklinik für Medizinische Psychologie und Psychotherapie, Auenbruggerplatz 3, A-8036 Graz, Tel.: +43 316 385 12516, eMail: christian.fazekas@medunigraz.at

---

Hiermit erkläre ich mich damit einverstanden, dass die durch mich im Rahmen der Studie übermittelten und sich auf meine Einrichtung beziehenden Antworten gespeichert, an der Universität Basel und dem Universitätsspital Basel pseudonymisiert ausgewertet, in anonymisierter Form an die anderen beteiligten Institutionen in Deutschland und Österreich weitergegeben werden und zur Beantwortung wissenschaftlicher Fragestellungen verwendet werden dürfen. Ausserdem bin ich damit einverstanden, dass die Daten in anonymisierter Form für wissenschaftliche Darstellungen und Veröffentlichungen verwendet werden dürfen. Ich nehme zur Kenntnis, dass ich die Teilnahme jederzeit ohne die Angabe von Gründen abbrechen kann.

☐ Ich bin darüber aufgeklärt worden, dass ich jederzeit die Teilnahme an der Studie beenden kann. Dies schliesst auch das Recht ein, meine Einwilligung in die Datenverarbeitung zu widerrufen. In diesem Falle wird der Personenbezug zu den Daten gelöscht.

- Es wurden folgende Ethikkommissionen konsultiert und entsprechend der gesetzlichen Vorgaben die notwendigen Ethikbewilligungen oder Nicht-

Zuständigkeitserklärungen eingeholt: Votum der Ethikkommissionen der Medizinischen Fakultät der Universität zu Köln (20-1416), Erklärung der Ethikkommission Nordwest- und Zentralschweiz (EKNZ) (Req-2020-00861).

· Ich wurde darüber informiert, dass die erhobenen Daten in pseudonymisierter Form gespeichert, weitergegeben und analysiert werden. Der Pseudonymisierungsprozess erfolgt, in dem die beantwortende Person gebeten wird, anstatt ihres Vollnamens, einen persönlichen Code zu generieren. Dieser Code dient als Schlüssel, der nur von der zu beantwortenden Person reproduzierbar ist, nicht jedoch – ohne unverhältnismässig grossen Aufwand – durch die Studienleitung oder durch Dritte.

· Als Person sind Sie in der Auswertung nicht erkennbar. Weiterhin wird Ihnen das Recht auf Auskunft und Berichtigung fehlerhaft verarbeiteter Daten eingeräumt. Sie haben das Recht, Einsicht in Ihre Daten zu nehmen, die während der Studie erhoben werden. Sollten Sie dabei Fehler in Ihren Daten feststellen, so haben Sie das Recht, diese über Prof. Dr. med. Rainer Schäfert korrigieren zu lassen. Falls Sie Fragen zu der Studie haben, können Sie sich an die unten angegebenen Ansprechpersonen wenden.

· Für Teilnehmende aus Deutschland und Österreich: Ich wurde informiert, dass meine Daten an ein Drittland – in diesem Fall in die Schweiz – ausserhalb der EU übermittelt werden, indem Sie die Daten direkt auf der Umfrageplattform eingeben und sich der Server in der Schweiz befindet (vgl. Art. 13 Abs. 1 lit. c und f DSGVO). Die Europäische Kommission hat in der Schweiz ein angemessenes gesetzliches Datenschutzniveau festgestellt (sog. Angemessenheitsbeschluss). Diesen Angemessenheitsbeschluss finden Sie unter https://edps.europa.eu/data-protection/data-protection/glossary/a_de.

· Wir sind grundsätzlich dazu verpflichtet, Ihnen kostenlos eine Kopie Ihrer personenbezogenen Daten zur Verfügung zu stellen. (Art. 15 Abs. 3 DSGVO).

· Sie haben das Recht, die zuständigen Datenschutzbeauftragen zu kontaktieren:

- Universitätsspital Basel: Danielle Kaufmann; eMail: danielle.kaufmann@unibas.ch oder Datenschutzbeauftragte@usb.ch.
- Medizinischen Universität Graz: Univ.-Prof. Dr. Thomas Wagner; eMail: datenschutz@medunigraz.at
- Universität Köln: 1. Datenschutzbeauftragter der Universität zu Köln: eMail: dsb@verw.uni-koeln.de; 2. Datenschutzbeauftragter der Uniklinik Köln: datenschutz@uk-koeln.de

· Sie haben auch das Recht, bei der zuständigen Aufsichtsbehörde für Datenschutz Beschwerde einzureichen: Die Landesbeauftragte für den Datenschutz Nordrhein-Westfalen, Kavalleriestrasse 2-4, 40213 Düsseldorf

**1.**	Wir möchten ermöglichen, dass Ihre Angaben auf Ihren Wunsch hin nachträglich gelöscht werden können, ohne Sie jedoch zu bitten, dazu Ihren Namen zu nennen. Daher bitten wir Sie, einen Code zu generieren, den nur Sie kennen und anhand dessen wir Ihren Datensatz bei Bedarf identifizieren können. Der Code besteht aus folgenden Elementen:
	1. Erster Buchstabe des Vornamens der Mutter<br>2. Erster Buchstabe des Vornamens des Vaters<br>3. Tag der Geburt der Mutter in zwei Ziffern (wenn 15.03.1948, dann 15)<br>4. Geburtsjahr der Mutter in zwei Ziffern (wenn 1948, dann 48)<br><br>Mein Code: _______________

**2.**	**Fragen zum Krankenhaus/Spital und zur Abteilung, auf die sich die Beantwortung dieser Umfrage bezieht:**
**2.a**	**Auf welches Krankenhaus/Spital bezieht sich die Beantwortung der Fragen?**
	1 Name des Krankenhauses/Spitals *(Freitext)*: ....…………………… ………..…<br>……………………………………………………………………………………...<br>2 Abteilung *(Freitext)*: ……………..………………………………………….…..……<br>………………………………………………………………………………………
**2.b**	**In welcher Stadt befindet sich Ihr Krankenhaus/Spital (überwiegend)?**
	(Freitext):<br>……………………………………..……………………………………………………<br>…

3.	**In welchem Staat und Bundesland bzw. Kanton befindet sich Ihr Krankenhaus/Spital?**	
	1 ☐ Deutschland	1.1 ☐ Baden-Württemberg<br>1.2 ☐ Bayern<br>1.3 ☐ Berlin<br>1.4 ☐ Brandenburg<br>1.5 ☐ Bremen<br>1.6 ☐ Hamburg<br>1.7 ☐ Hessen<br>1.8 ☐ Mecklenburg-Vorpommern<br>1.9 ☐ Niedersachsen<br>1.10 ☐ Nordrhein-Westfalen<br>1.11 ☐ Rheinland-Pfalz<br>1.12 ☐ Saarland<br>1.13 ☐ Sachsen<br>1.14 ☐ Sachsen-Anhalt<br>1.15 ☐ Schleswig-Holstein<br>1.16 ☐ Thüringen
	2 ☐ Österreich	2.1 ☐ Vorarlberg<br>2.2 ☐ Tirol<br>2.3 ☐ Salzburg<br>2.4 ☐ Kärnten<br>2.5 ☐ Steiermark<br>2.6 ☐ Oberösterreich<br>2.7 ☐ Niederösterreich<br>2.8 ☐ Burgenland<br>2.9 ☐ Wien

	[3] ☐ Schweiz	[2.1] ☐ Aargau<br>[2.2] ☐ Appenzell Innerhoden<br>[2.3] ☐ Appenzell Ausserhoden<br>[2.4] ☐ Bern<br>[2.5] ☐ Basel Land<br>[2.6] ☐ Basel Stadt<br>[2.7] ☐ Freiburg<br>[2.8] ☐ Genf<br>[2.9] ☐ Glarus<br>[2.10] ☐ Graubünden<br>[2.11] ☐ Jura<br>[2.12] ☐ Luzern<br>[2.13] ☐ Neuenburg<br>[2.14] ☐ Nidwalden<br>[2.15] ☐ Obwalden<br>[2.16] ☐ St.Gallen<br>[2.17] ☐ Schaffhausen<br>[2.18] ☐ Solothurn<br>[2.19] ☐ Schwyz<br>[2.20] ☐ Thurgau<br>[2.21] ☐ Tessin<br>[2.22] ☐ Uri<br>[2.23] ☐ Waadt<br>[2.24] ☐ Wallis<br>[2.25] ☐ Zug<br>[2.26] ☐ Zürich

**4.**	**In was für einem Krankenhaus/Spital sind Sie tätig?**<br>*Bitte wählen Sie nur eine der folgenden Antworten aus (Einfachauswahl):*
	[1] ☐ Universitätsklinikum/Universitätsspital<br>[2] ☐ Allgemeinklinik<br>[3] ☐ Fachklinik<br>[4] ☐ Belegkrankenhaus<br>[5] ☐ Andere Krankenhausart, wenn ja, welche *(Freitext)*:<br>……………………………………………………..…

5.	**In welcher Trägerschaft befindet sich Ihr Krankenhaus/Spital?**<br>*Bitte wählen Sie nur eine der folgenden Antworten aus (Einfachauswahl).*
	[1] ☐ Privater Träger<br>[2] ☐ Freigemeinnütziger Träger (z. B. kirchliche Träger)<br>[3] ☐ Öffentlicher Träger (inkl. öffentlich-rechtliche Institutionen / Aktiengesellschaft in staatlichem Besitz etc.)<br>[4] ☐ Andere Trägerschaft

6.	**Über wie viele Betten verfügt Ihr Krankenhaus/Spital insgesamt?**
	________ (*Angabe Zahl*)

7.	**Aus welcher primären fachlichen Perspektive beantworten Sie diese Umfrage?**<br>*(Mehrfachantwort möglich)*
	[1] ☐ Psychosomatik<br>[2] ☐ Psychiatrie<br>[3] ☐ Kinder- und Jugendpsychiatrie und -Psychosomatik<br>[4] ☐ Medizinische Psychologie<br>[5] ☐ Psychologischer Dienst / Psychologische Abteilung<br>[6] ☐ andere, wenn ja, welche *(Freitext)*:<br>…………………………………………………………………………..

8.	**Welche psychosozialen Dienste gibt es in Ihrem Krankenhaus/Spital?**<br>*(Mehrfachantwort möglich)*
	[1] ☐ Psychosomatischer Konsiliar-/ Liaison-Dienst<br>[2] ☐ Psychiatrischer Konsiliar-/ Liaison-Dienst<br>[3] ☐ Psychologischer Konsiliar-/ Liaison-Dienst<br>[4] ☐ andere, wenn ja, welche (Freitext):<br>……………………………………………………………………………..…<br>………………………………………………………………………..………

**9.**	**Über wie viele Vollzeitstellen verfügt der Konsiliar-/Liaison-Dienst Ihrer eigenen Abteilung/Klinik? (***jeweils Antwort in Form einer Zahl)*
	1 ☐ Ärzt*innen-Stellen: ____<br>2 ☐ Psycholog*innen-Stellen: ____<br>3 ☐ Sozialarbeiter*innen-Stellen: ____<br>4 ☐ Pflegefachpersonen: ____<br>5 ☐ andere Stellen-Anteile: ____

**10.**	**Einbindung und Auslastung Ihres Krankenhauses/Spitals in die *somatische* Versorgung von COVID-19 Patient*innen seit Beginn der Pandemie.**
**10.a**	**Seit Beginn der Pandemie, wie stark war/ist Ihr Krankenhaus/Spital in die *somatische* Versorgung von COVID-19 Patient*innen maximal eingebunden?**
	**0 – 1 – 2 – 3 – 4 – 5**<br>**(0 = überhaupt nicht bis 5 = sehr stark)**
**10.b**	**Wie stark war/ist Ihr Krankenhaus/Spital hinsichtlich der *somatischen*Versorgung von COVID-19 Patient*innen maximal ausgelastet?**
	**0 – 1 – 2 – 3 – 4 – 5**<br>**(0 = überhaupt nicht bis 5 = sehr stark)**

**11.**	**Wo wurden/werden COVID-19-Patient*innen stationär in Ihrem Krankenhaus/Spital behandelt?** *(Mehrfachantwort möglich)*
	1 ☐ speziell ausgewiesene Station(en) für Verdachtsfälle<br>2 ☐ speziell ausgewiesene Station(en) für COVID-19-Patient*innen<br>3 ☐ speziell ausgewiesene Intensivstation(en)/-betten<br>4 ☐ sonstige

12.	**Wurden in Ihrem Krankenhaus/Spital weitere spezielle Strukturen zur somatischen Versorgung im Zusammenhang mit COVID-19 etabliert?**
	1 ☐ nein<br>2 ☐ ja und zwar *(Mehrfachantwort möglich)*:
	2.1 ☐ Zusätzliche Intensivmedizinische Behandlungsplätze
	2.2 ☐ Zusätzliche Beatmungsplätze
	2.3 ☐ Umwandlung regulärer Stationen/Bereiche zur Behandlung von COVID19 Patienten
	2.4 ☐ Umwandlung regulärer Stationen/Bereiche zur Abklärung / Behandlung von COVID19 Verdachtsfällen
	2.5 ☐ Ambulantes Abklärungs-/Testzentrum auf SARS-CoV-2/COVID-19
	2.6 ☐ Ausbau spezifischer Laborkapazitäten zur Testung auf SARS-CoV-2

13.a	**Ist bei Ihnen eine psychosoziale Versorgung im Zusammenhang mit COVID-19 erfolgt?**
	1 ☐ Bei uns ist keine COVID-19 bezogene Versorgung erfolgt.<br>2 ☐ Bei uns ist eine COVID-19 bezogene Versorgung erfolgt.
**13.b**	**Für den Fall, dass bei Ihnen eine psychosoziale Versorgung im Zusammenhang mit COVID-19 erfolgt (ist): Wie verteilt/e sich die zeitliche Inanspruchnahme dieser Versorgung durch das psychosoziale Team Ihrer Klinik/Abteilung während Zeiten höchster Beanspruchung prozentual grob geschätzt auf die drei Zielgruppen** *(soll sich insgesamt auf 100% aufsummieren)*?
	Patient*innen: _____%<br>Angehörige: _____%<br>Mitarbeitende: _____%<br>**Gesamt: 100 %**

14.	**Wurden Kooperationsstrukturen innerhalb des Krankenhauses/Spitals zur psychosozialen Unterstützung im Kontext der COVID-19-Pandemie aufgebaut?**
	1 ☐ nein<br>2 ☐ ja – falls ja, wer war beteiligt? (*Mehrfachantwort möglich*)
	3 ☐ Psychosomatik<br>4 ☐ Psychiatrie<br>5 ☐ Palliativmedizin<br>6 ☐ Psychologie/Medizinpsychologie<br>7 ☐ Psychoonkologie<br>8 ☐ Seelsorge<br>9 ☐ Sozialdienst<br>10 ☐ Pflege<br>11 ☐ sonstige *(Freitext)*:<br>………………..……………………………………………...…………..

15.	**Welche Strukturen zur psychosozialen Versorgung in der Corona-Pandemie wurden in Ihrem Krankenhaus/Spital entwickelt?** *(Mehrfachantwort möglich)*
	1 ☐ Es wurden neue Versorgungsstrukturen etabliert.<br>2 ☐ Bereits bestehende Versorgungsstrukturen wurden weiterentwickelt.<br>3 ☐ Die Versorgungsstruktur blieb unverändert.<br>4 ☐ Es war/ist keine psychosoziale Versorgung möglich.
	Kommentar *(optional)*:<br>…………………………………………………………………………………

16.	**Wurde für psychosoziale Themen im Pandemie-Krisenstab Ihres Krankenhauses/ Spitals ein*e spezifische Vertreter*in bestimmt?**
	[1] ☐ nein<br>[2] ☐ ja und zwar: ***(**Mehrfachantwort möglich)*
	[3] interne Person: Bestimmung einer zuständigen Person aus den psychosozialen Abteilungen<br>☐ interne Person: regelmässige Teilnahme an Sitzungen des Pandemie-Krisenstabs<br>☐ interne Person: punktuelle Teilnahme an Sitzungen des Pandemie-Krisenstabs
	[4] externe Person: Bestimmung einer zuständigen Person von ausserhalb der psychosozialen Abteilungen<br>☐ externe Person: regelmässige Teilnahme an Sitzungen des Pandemie-Krisenstabs<br>☐ externe Person: punktuelle Teilnahme an Sitzungen des Pandemie-Krisenstabs
	Kommentar *(optional)*:<br>………………………………………………………………………………

17.	**Wie war/ist die psychosoziale Versorgung für COVID-19-Patient*innen zeitlich <u>maximal</u> verfügbar?** *(Mehrfachauswahl)*
	[1] ☐ täglich, je 24 Stunden<br>[2] ☐ täglich, von 8 bis 24 Uhr<br>[3] ☐ täglich, zu den üblichen Arbeitszeiten, z. B. 8 bis 17 Uhr<br>[4] ☐ Montag bis Freitag, je 24 Stunden<br>[5] ☐ Montag bis Freitag, zu den üblichen Arbeitszeiten, z. B. 8 bis 17 Uhr<br>[6] ☐ weniger als 5 ausgewählte Tage pro Woche<br>[7] ☐ Zusätzlich zu den oben genannten Zeiten gab es eine Rufbereitschaft oder Bereitschaftsdienst.<br>[8] ☐ sonstiges – wie? (Freitext):<br>…………………………………………………………………………………<br>………………………………………………………………

**18.**	**Wurden im COVID-19-Kontext in Ihrem Krankenhaus/Spital spezifische Angebote oder Vorgehensweisen zur psychosozialen Unterstützung <u>für Patient*innen</u> etabliert?**	**Inwieweit haben sich diese Vorgehensweisen/Angebote bewährt?**<br>**0 – 1 – 2 – 3 – 4 – 5**<br>**(0 = überhaupt nicht**<br>**bis 5 = sehr stark)**
	1 ☐ nein<br>2 ☐ ja – Falls ja, welche? *(Mehrfachantwort möglich)*	
	3 spezifische Vorgehensweisen / *Standard Operation Procedures (SOPs)* für häufige Fragestellungen	**0 – 1 – 2 – 3 – 4 – 5**
	4 Konsil-Mitarbeitende speziell für Corona-Anfragen	**0 – 1 – 2 – 3 – 4 – 5**
	5 Liaison-Mitarbeitende auf COVID-Stationen	**0 – 1 – 2 – 3 – 4 – 5**
	6 psychosoziales Corona-Care-Team	**0 – 1 – 2 – 3 – 4 – 5**
	7 telefonische Corona-Hotline	**0 – 1 – 2 – 3 – 4 – 5**
	8 ambulante Corona-Sprechstunde	**0 – 1 – 2 – 3 – 4 – 5**
	9 Nachsorge-Angebote für Patient*innen mit post-COVID-Syndrom	**0 – 1 – 2 – 3 – 4 – 5**
	10 ☐ sonstiges – was? (Freitext):<br>……………………………………………………………<br>……………………………………………………………<br>……………………………...…………………………….<br>……………………………………………………………	**0 – 1 – 2 – 3 – 4 – 5**

19.	**Wurden im COVID-19-Kontext in Ihrem Krankenhaus/Spital spezifische Angebote oder Vorgehensweisen zur psychosozialen Unterstützung <u>für Angehörige</u> etabliert?**	**Inwieweit haben sich diese Vorgehensweisen/ Angebote bewährt?<br>0 – 1 – 2 – 3 – 4 – 5<br>(0 = überhaupt nicht<br>bis 5 = sehr stark)**
	1 ☐ nein<br>2 ☐ ja - Falls ja, welche? *(Mehrfachantwort möglich)*	
	3 ☐ spezifische Vorgehensweisen / Standard Operation Procedures (SOPs) für die Unterstützung von Angehörigen	**0 – 1 – 2 – 3 – 4 – 5**
	4 ☐ Telefonische Corona-Hotline auch für Angehörige	**0 – 1 – 2 – 3 – 4 – 5**
	5 ☐ spezifisches Beratungs- und Gesprächs-Angebot für Angehörige	**0 – 1 – 2 – 3 – 4 – 5**
	6 ☐ sonstiges – was? (Freitext):<br>………………………………………………………………<br>………………………………………………………………<br>………………………………………………………………<br>………………………………………………………………<br>……………………..…………………………………………..	**0 – 1 – 2 – 3 – 4 – 5**

20.	**Wurden im COVID-19-Kontext in Ihrem Krankenhaus/Spital spezifische Angebote oder Vorgehensweisen zur psychosozialen Unterstützung <u>für Mitarbeitende</u> etabliert?**	**Inwieweit haben sich diese Vorgehensweisen/ Angebote bewährt?<br>0 – 1 – 2 – 3 – 4 – 5<br>(0 = überhaupt nicht<br>bis 5 = sehr stark)**
	1 ☐ nein<br>2 ☐ ja - Falls ja, welche? *(Mehrfachantwort möglich)*	

	[3] ☐ Fallbesprechungen zu belastenden Patienten-Situationen	**0 – 1 – 2 – 3 – 4 – 5**
	[4] ☐ Team-Supervision / moderierter Gruppenaustausch zum Erleben der Corona-Situation als Mitarbeitende & im Team	**0 – 1 – 2 – 3 – 4 – 5**
	[5] ☐ Sprechstunden-/Beratungsangebot für Mitarbeitende	**0 – 1 – 2 – 3 – 4 – 5**
	[6] ☐ Telefon-Hotline für Mitarbeitende	**0 – 1 – 2 – 3 – 4 – 5**
	[7] ☐ gezielte Arbeit mit Führungspersonen bezüglich hilfreicher Unterstützungs-Massnahmen für das Personal/ die Teams	**0 – 1 – 2 – 3 – 4 – 5**
	[8] ☐ Schulung zum Umgang mit psychosozialen Belastungen von Patient*innen und Angehörigen (Erkennen, Kommunikation, Umgang)	**0 – 1 – 2 – 3 – 4 – 5**
	[9] ☐ Workshops zur Stärkung der Resilienz der Mitarbeitenden (z. B. Selbstfürsorge/ Ressourcenaktivierung, „Psychohygiene“)	**0 – 1 – 2 – 3 – 4 – 5**
	[10] ☐ Schaffung von Ruhemöglichkeiten in hochbelasteten Teams	**0 – 1 – 2 – 3 – 4 – 5**
	[11] ☐ sonstiges – was? (*Freitext*):<br>………………………………………………………………<br>………………………………………………………………<br>………………………………………………………………<br>………………………………………………………………<br>………………………………………………………………	**0 – 1 – 2 – 3 – 4 – 5**

**21.**	**Kommunikation: Wie wurden/werden die verschiedenen Zielgruppen über die COVID-19 bezogenen Angebote zur psychosozialen Unterstützung informiert?** *(Mehrfachantwort möglich)*
**21.a**	**Patient*innen:**
	1 ☐ Flyer<br>2 ☐ Aushang<br>3 ☐ Internet<br>4 ☐ persönlich<br>5 ☐ Screening<br>6 ☐ Mund-zu-Mundpropaganda<br>7 ☐ keine speziellen Massnahmen<br>8 ☐ andere, wenn ja, welche *(Freitext)*:<br>……………………………………………………………...
**21.b**	**Angehörige:**
	1 ☐ Flyer<br>2 ☐ Aushang<br>3 ☐ Internet<br>4 ☐ persönlich<br>5 ☐ Mund-zu-Mundpropaganda<br>6 ☐ keine speziellen Massnahmen<br>7 ☐ andere, wenn ja, welche *(Freitext)*:<br>……………………………………………………………...
**21.c**	**Mitarbeitende:**
	1 ☐ Flyer<br>2 ☐ Aushang<br>3 ☐ Internet<br>4 ☐ persönlich<br>5 ☐ Mund-zu-Mundpropaganda<br>6 ☐ Information über Führungskräfte<br>7 ☐ keine speziellen Massnahmen<br>8 ☐ andere, wenn ja, welche *(Freitext)*:<br>…...…………………………………………………………...

**22.**	**Wie schätzen Sie die maximale Belastung Ihres eigenen psychosozialen Teams Ihrer Klinik/Abteilung durch die COVID-19-Pandemie ein?**			
**22.a**	**auf einer Skala 0 – 10 (0 = gar nicht belastet bis 10 = extrem belastet)**			
	**gar nicht belastet**	**0 ——————————————10**		**extrem belastet**
**22.b**	**In welchem Zeitraum war die Belastung am höchsten?**<br><br>**Bitte geben Sie den Zeitraum in folgendem Format an: Bsp. 01/2001**			
	**von (Monat/Jahr)**		**bis (Monat/Jahr)**	

Bedarf und Wünsche für die Zukunft:

**23.**	**Haben Sie oder Ihr Team Wünsche nach Austausch/Unterstützung bezüglich der psychosozialen Versorgung im COVID-19-Kontext?**
	[1] ☐ nein<br>[2] ☐ ja – falls ja, welche? *(Mehrfachantwort möglich)*
	[3] ☐ Erfahrungsaustausch mit anderen Kliniken<br>[4] ☐ Austausch zu spezifischen Vorgehensweisen / Standard Operation Procedures (SOPs) für häufige Fragestellungen<br>[5] ☐ Austausch zu Versorgungsstrukturen<br>[6] ☐ Austausch zur Mitarbeiter-Unterstützung<br>[7] ☐ Austausch zur Angehörigen-Unterstützung<br>[8] ☐ Hospitation in einer anderen Klinik<br>[9] ☐ Fortbildungen zu psychosozialen Themen im Kontext COVID-19<br>[10] ☐ sonstiges – falls ja, welche? *(Freitext)*:<br>……………………………………………………………………………<br>……………………………………………………………………………<br>……………………………………………………………………………

24.	**Gibt es Änderungen/Verbesserungen, die Sie für die Zukunft in Bezug auf die psychosozialen Versorgungsangebote in Ihrem Krankenhaus/Spital im COVID-19-Kontext für essentiell halten?**
	1 ☐ nein<br>2 ☐ ja – falls ja, welche? *(Mehrfachantwort möglich)*
	3 ☐ bezüglich Versorgungsstrukturen<br>4 ☐ bezüglich abteilungsübergreifender Zusammenarbeit<br>5 ☐ bezüglich interprofessioneller Zusammenarbeit<br>6 ☐ bezüglich bereitgestelltem Personal<br>7 ☐ bezüglich Informationsprozessen (Bekanntmachung von Angeboten, etc.)<br>8 ☐ bezüglich Angeboten zur Entlastung des Konsiliar-Liaison-Dienst-Teams<br>9 ☐ sonstige – falls ja, was? *(Freitext)*:<br>………………………………………………………………………………<br>………………………………………………………………………………<br>………………………………………………………………………………

25.	**Gibt es im COVID-19-Kontext aus Ihrer Sicht weitere Aspekte, die für den Konsiliar-Liaison-Dienst von Bedeutung sind und die Sie ergänzen möchten?**
	1 ☐ nein<br>2 ☐ ja
	Falls ja, welche *(Freitext)*:

Vielen Dank für Ihre Beteiligung an dieser Umfrage!
Bei Fragen wenden Sie sich bitte an eMail: covid-survey-medizin@unibas.ch
